# Electron microscopy imaging and mechanical characterization of T47D multicellular tumor spheroids–Older spheroids reduce interstitial space and become stiffer

**DOI:** 10.1371/journal.pone.0286291

**Published:** 2023-05-25

**Authors:** Mathangi Madhavan, Devina Jaiswal, Sarah Karlberg, Alexis Duggan, Hassan A. Almarshad, Kevin P. Claffey, Kazunori Hoshino

**Affiliations:** 1 Department of Biomedical Engineering, University of Connecticut, Storrs, Connecticut, United States of America; 2 Department of Biomedical Engineering, Western New England University, Springfield, Massachusetts, United States of America; 3 College of Applied Medical Sciences, Al Jouf University, Sakakah, Al Jawf, Saudi Arabia; 4 Department of Cell Biology, University of Connecticut Health Center, Farmington, Connecticut, United States of America; Technion-Israel Institute of Technology, ISRAEL

## Abstract

Multicellular cancer spheroids are an *in vitro* tissue model that mimics the three-dimensional microenvironment. As spheroids grow, they develop the gradients of oxygen, nutrients, and catabolites, affecting crucial tumor characteristics such as proliferation and treatment responses. The measurement of spheroid stiffness provides a quantitative measure to evaluate such structural changes over time. In this report, we measured the stiffness of size-matched day 5 and day 20 tumor spheroids using a custom-built microscale force sensor and conducted transmission electron microscopy (TEM) imaging to compare the internal structures. We found that older spheroids reduce interstitial spaces in the core region and became significantly stiffer. The measured elastic moduli were 260±100 and 680±150 Pa, for day 5 and day 20 spheroids, respectively. The day 20 spheroids showed an optically dark region in the center. Analyzing the high-resolution TEM images of spheroid middle sections across the diameter showed that the cells in the inner region of the day 20 spheroids are significantly larger and more closely packed than those in the outer regions. On the other hand, the day 5 spheroids did not show a significant difference between the inner and outer regions. The observed reduction of the interstitial space may be one factor that contributes to stiffer older spheroids.

## 1. Introduction

Monolayer cell culture does not adequately represent the three-dimensional (3D) structural and spatial heterogeneity in the tumor microenvironment, which is believed to contribute to the mechanisms of cancer cell growth, tumor metastasis, and drug responses [1–5]. On the other hand, *in vivo* tumor models with immune-deficient mouse models can be expensive and require specialized animal facilities. They also exhibit additional complications such as metabolic mechanisms, which are different for humans and mice. There is evidence of change in cellular behavior in 3D cultures, which can alter the differentiation capability of stem cells [6] and genetic modification in cancer cells [7]. The use of multicellular spheroids is an approach to fill the technological gap between monolayer cultures and animal models [8]. It is believed that 3D multicellular spheroids better mimic the physiological cell-cell and cell-extracellular matrix interaction than the conventional 2D monolayer cultures [9]. Multicellular spheroids have been used as a platform to test drug delivery systems [2, 8], study molecular changes [10, 11], and form 3D structures [12, 13]. A comparative study conducted on 2D and 3D cultured breast cancer cell lines showed that 3D cultured cells developed an increased resistance to cancer drugs with a higher number of dormant cells compared to cells cultured as monolayers [14]. Spheroids are believed to promote cell-cell interaction which can directly affect cellular growth, metabolism and cell response mediated by signaling pathways [15]. Cell-cell interaction in 3D culture may affect the downstream signaling leading to cell response.

The cellular arrangement can also affect the structural characteristics of a spheroid. The study made by Kenny *et al*. comparing multiple breast cancer cell lines showed changes in cellular arrangement and polarity when cultured as multicellular spheroids, which impacts the microenvironmental signals received by the adhesion molecules [16]. Drug response studies can be susceptible to this variability. Image analysis of the internal structure of spheroids has been made to evaluate the effect of size and volume of spheroids on drug response [17]. *In vitro* multicellular spheroids exhibit the gradient nutrient and metabolic waste distribution which can lead to development of distinct zones that can be categorized as proliferating, quiescent and necrotic core [8]. Cellular response to drugs in each zone can be different, which mimics the cellular make up of physiological tumors that exhibit limited mass transport to the entire tumor [18]. As spheroids grow, they develop the gradients of oxygen, nutrients, and catabolites. In particular, the study of oxygen dynamics is highly relevant for the design of new anticancer therapeutics [19–23]. In the hypoxic region, the efficacy of drugs that promote cellular deaths through the formation of reactive oxygen species is reduced. Drugs that are effective in proliferative cells do not work sufficiently for senescent and necrotic cells in the hypoxic region [20]. The existence of hypoxic cells that are resistant to radiation can cause failure in radiation therapy. Multicellular tumor spheroids also serve as a model to study the effects of radiation by better mimicking the poorly oxygenated areas in tumors [21].

The growth of spheroids is categorized into three successive phases: exponential, linear, and plateau [20, 21]. The first phase corresponds to early spheroid formation and proliferation [21]. At the initial stage, the volume of a spheroid increases exponentially. The volume growth rate then decreases and eventually reaches a plateau [20, 21, 23]. The linear and plateau phases represent the formation of a non-proliferative inner region and a necrotic center, respectively [21]. Cells in the periphery show high proliferation because they have access to oxygen and nutrients. On the other hand, cells in the inner region stay in a senescent or necrotic state [20]. Larger spheroids are more prone to develop secondary central necrosis, where some of the internal cells die [24].The growth curves are often empirically approximated by logistic and Gompertzian sigmoid functions. There have also been approaches to developing an analytical model that addresses the underlying processes of oxygen dynamics and spheroid growth [22]. Grimes *et al*. used a diffusion equation and estimated the local oxygen partial pressure, diffusion limit, necrotic core formation, hypoxic region, and proliferating rim [23]. They derived a time-dependent growth model for tumor spheroids using oxygen diffusion, oxygen consumption rate, and average cellular doubling time as free parameters [22]. It is difficult to evaluate oxygen distribution in solid tumors in vivo because of the complexity of vasculature patterns and flow dynamics in tumors. In vitro analytical models are useful for estimating the formation of the anoxic, hypoxic and viable regions and determining the oxygen consumption rate [22].

Mechanical characterization is an engineering approach to quantifying the properties of materials. Also, for cancer studies, the mechanical properties of the tissue can be regarded as a biomarker for tumor phenotyping. Techniques such as atomic force microscopy [25], optical tweezers [26], and magnetic tweezers [27] have been used to mechanically characterize single cancer cells, whereas tissue indentation techniques have been used to mechanically characterize cancer tissues [28]. However, these techniques do not allow one to fully evaluate cell-cell mechanical interaction in micro/meso-scale (100 μm to 1 mm) tissues such as cellular spheroids. In our previous study, we developed unique micro-mechanical tweezers and demonstrated mechanical characterization of breast cancer spheroids and normal epithelial cell spheroids [29]. Here we utilize this unique mechanical testing capability and quantify the structural changes in growing spheroids. We measure the mechanical elastic moduli of size-matched day 5 and day 20 spheroids.

In order to correlate the stiffness changes with the cellular-scale morphological changes, we obtained high-resolution transmission electron microscopy (TEM) images of spheroid middle sections across the diameter and conducted a detailed image analysis to evaluate the difference in the internal cellular organization. TEM imaging provides a resolution much higher than optical microscopes and is suitable for quantitative analysis. On the other hand, measuring the fluorescence intensity may be influenced by many parameters.

## 2. Materials and methods

### 2.1 Spheroid preparation

The human mammalian epithelial breast cancer cell line (T47D) was used for the study (American Type Culture Collection, Manassas, VA). We used Dulbecco’s Modified Eagle’s Medium (DMEM) added with 10% of Fetal Bovine Serum (FBS, Fisher Scientific, USA) and 1% Penicillin-Streptomycin (Gibco, Fisher Scientific, USA). The spheroids were prepared and grown on the 96-well plate (Corning, NY, USA) surface treated with 50 μl of agarose in each well. The monolayer cancer cells in 60 mm petri dish were trypsinized and counted in a hemocytometer (Hausser Scientific, Horsham, PA, USA). In this study, two differently aged, size-matched spheroids were cultured; day 5 and day 20, respectively. The day 5 spheroids were seeded on a 96 U bottom well plate with a density of 4000 cells/well, and the day 20 spheroids were seeded at a density of 1000 cells/ well. The spheroids were then incubated under standard conditions of 37˚C temperature and 5% CO_2_ in humidified incubators. The size characterization of the spheroids was evaluated with the help of ImageJ software. We used the live/dead viability/cytotoxicity kit (Thermo Fisher, MA, USA) for the cell viability assay.

### 2.2 Stiffness characterization

The method for stiffness analysis using microtweezers has been explained previously [29]. Briefly, T47D spheroids (day 20 and day 5, size matched) were placed in a shallow well with media. The micro-cantilevers (L: 1.6 mm, w: 100 μm, t = 15 μm) were lowered into the well. A voltage input of 1.1 V/step from 0 V to typically ~40 V was given to the piezo actuator, which produced an average displacement of 4.9± 0.5 μm/step at the tweezer tip. An additional reference experiment was conducted for each experiment, where tweezers were operated without the spheroid. The optical images obtained for each compression were analyzed using custom-built pattern recognition MATLAB software. COMSOL was used to estimate the Young’s modulus for each measurement by modeling the spheroid as a sphere.

### 2.3 TEM analysis

TEM (FEI Tecnai 12 G2 Spirit BioTWIN) was used for sectioned images. The cultured T47D breast cancer epithelial spheroids were processed for TEM at the Biosciences Electron Microscopy Laboratory (UConn). The sample preparation included fixation, agarose 31 embedding, oxidation, dehydration, and embedment of the spheroid samples as well as ultramicrotomy.

#### Sample preparation

The day 5 (4000 cells/ well) and day20 (1000cells/well) spheroids were transferred to an Eppendorf tube prepared for each condition. The cells were rinsed with PIPES buffer and later the buffer was removed and immediately replaced with 1,500 μl EM fixative (2.5% glutaraldehyde + 4.0% paraformaldehyde in 0.1M PIPES + 2 mM CaCl_2_ + 4 mM MgCl_2_, pH 6.8). Later the samples were fixed overnight at 4°C. Ultra-low gelling temperature agarose (3%) was melted and held at ~42°C in a water bath. The fixatives from the Eppendorf tubes were removed carefully using micropipettes. A Layer of 500 μl of liquefied 3% agarose was added on top of the cell suspension for each condition and was allowed to solidify for 1 hour in a tube that was in an ice bucket. The agarose pellet containing the tissue was cut into ~1 mm pieces, to be treated as tissue blocks, and rinsed five times in 0.1 M PIPES buffer (pH 6.8) for 10 minutes each. Reduced osmium postfixation was conducted in 1% osmium tetroxide and 1.5% potassium ferrocyanide, and PIPES buffer for 2 hours. The tissue was rinsed twice in PIPES buffer for 10 minutes each followed by a rinse in distilled water. The tissue was incubated in thiocarbohydrazide for 30 minutes and rinsed in distilled water. Secondary fixation was conducted in 1% osmium tetroxide for 1 hour, and rinsed in distilled water. The tissue was incubated in PIPES buffer overnight. The following day, the tissue was rinsed in PIPES buffer for 10 minutes, then tissue was fixed in 2% aqueous uranyl acetate for 2 hours, and rinsed in distilled water three times. Lead aspartate fixation was conducted for 1 hour in the oven at 60°C. Prior to dehydration, tissue was rinsed in distilled water. Tissue was dehydrated through a series of graded ethanol (50%, 70%, 95%, and 100% twice), ethanol-acetone mixture (1:1 and 2:1), and acetone (three times) for 10 minutes each. Epon resin containing LX-112, nadic methyl anhydride (NMA), dodecenyl succinic anhydride (DDSA) and dimethyl aminoethyl phenol (DMP-30) were freshly prepared. The tissue was infiltrated in graded mixtures of epon resin:acetone for 24 hours each (1:1 and 3:1). Samples were then infiltrated in 100% epon resin for 6 hours with one change of resin after 3 hours. Tissue was placed and oriented in flat double end molds (Ted Pella, Inc.) and polymerized in an oven under a vacuum at 60°C for 48 hours.

#### Serial sectioning

Semi-thin sections (~ 1 μm) were cut with a histo 45° Diatome™ diamond knife on a Leica Ultracut UCT microtome and collected on drops of distilled water on Superfrost® Plus microscope slides (Fisher Scientific) and allowed to dry on a 30–8010 AB slide warmer (Buehler Ltd). Sections were stained with a working solution of 1:1 methylene blue:azure blue II in the slide warmer for 15 seconds at 80°C. Finally, sections were examined at the light microscope level in an Olympus microscope to identify suitable material for electron microscopy. We chose the area that contained the largest part of a spheroid to obtain a TEM sample at the middle section. Ultrathin (~ 100 nm) sections were cut with an ultra 45° Diatome™ diamond knife on a Leica Ultracut UCT microtome, decompressed with trichloroethylene, and collected on pioloform coated 2 × 1 mm copper/berilium slot grids. Images were obtained using a bright field FEI Tecnai Biotwin G2 Spirit (Netherlands) transmission electron microscope operated at an accelerating voltage of 80 kV and equipped with an AMT 2k (4 megapixels) XR40 CCD camera. The primary axis (i.e. the longest distance) of the spheroid’s cross-sectional ellipse was used in stitched high-resolution TEM imaging. The tracking tool for the TEM microscope was enabled to track the xy coordinate and obtain TEM images along the selected primary axis with sufficient overlap.

### 3. Result

#### 3.1 Stiffness characterization

**Fig 1:**
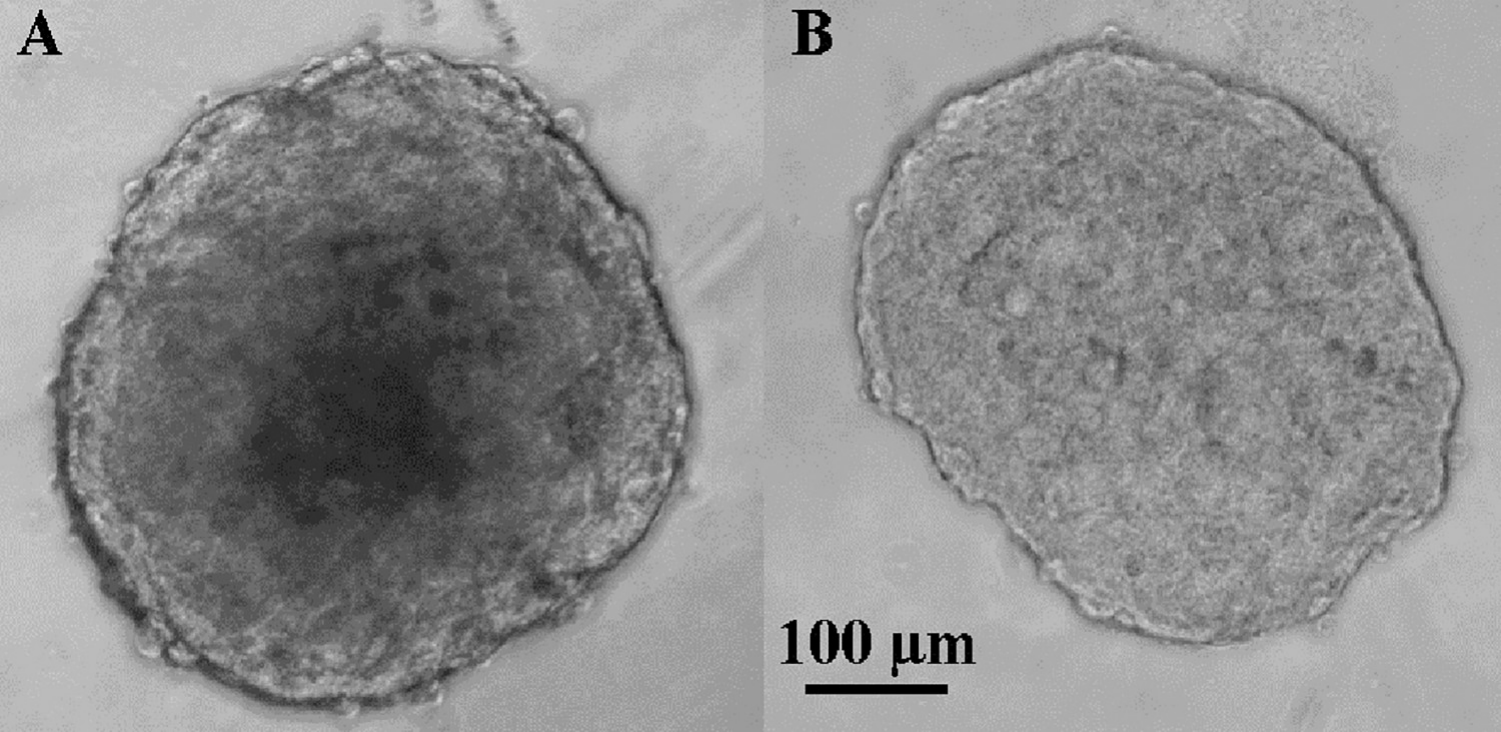
Example of (A) day 20 and (B) size-matched day 5 T47D spheroid. A dense center is visible in the day 20 spheroid. Day 20 spheroids were seeded from 1000 cells/ well and day 5 spheroids were seeded from 4000 cells/well to prepare size-matched samples.

**Fig 2:**
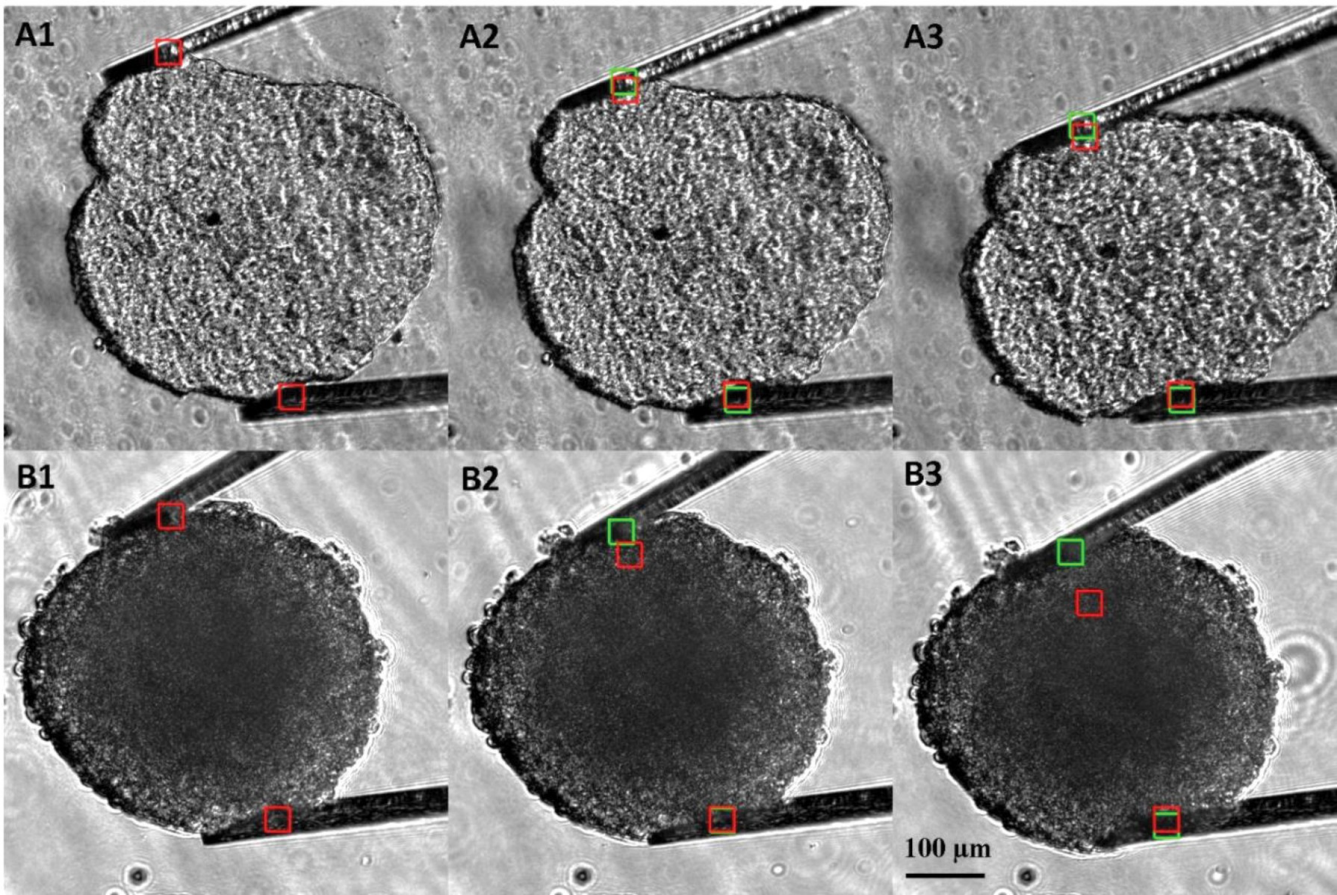
Mechanical characterization of T47D day 5 (Panel A) and day 20 (Panel B) was preformed using microtweezers. The average size of spheroids was 425 μm. Day 20 spheroids exerted higher force on the microcantilevers than day 5, size matched, spheroids.

We observed the growth of distinct necrotic centers in older spheroids, which has been reported [8, 17]. Fig 1 shows the difference in spheroid core with respect to time in day 20 (Fig 1A) and day 5 (Fig 2B,). The average sizes of T47D spheroids at day 20 and day 5, respectively, were 457±16 μm and 421±26 μm. Mechanical analysis of day 5 and day 20 spheroids was conducted for n = 6 samples using microtweezers. Fig 2 shows the compression steps of T47D day 5 (Fig 2, Panel A) and day 20 (Fig 2, Panel B) spheroids. The red squares indicate the reference positions of the cantilever tips measured without a spheroid, and the green square depicts the position of cantilever tips when the spheroid is compressed between the cantilevers. The colored squares were generated from the custom-built pattern matching software (see [29] for details). Comparing sequences A1-A3 and B1-B3 in Fig 2, one can see that the separation between the top green square and its respective red square is greater in B rather than in A. This difference shows that the cantilevers in B1-B3 were bent more than in A1-A3 because the spheroid in B1-B3 (i.e. day 20 spheroid) is stiffer than the one in A1-A3 (i.e. day5 spheroid). We found that day 20 spheroids exert ~ 2.5 times more force on average than day 5 spheroids. The average Young’s moduli of T47D day 5 and day 20 spheroid were 260±100 and 680±150 Pa, respectively, which shows day 20 spheroids are significantly stiffer (p<0.0005) than day 5 spheroids.

### 3.2 Transmission electron microscopy

**Fig 3:**
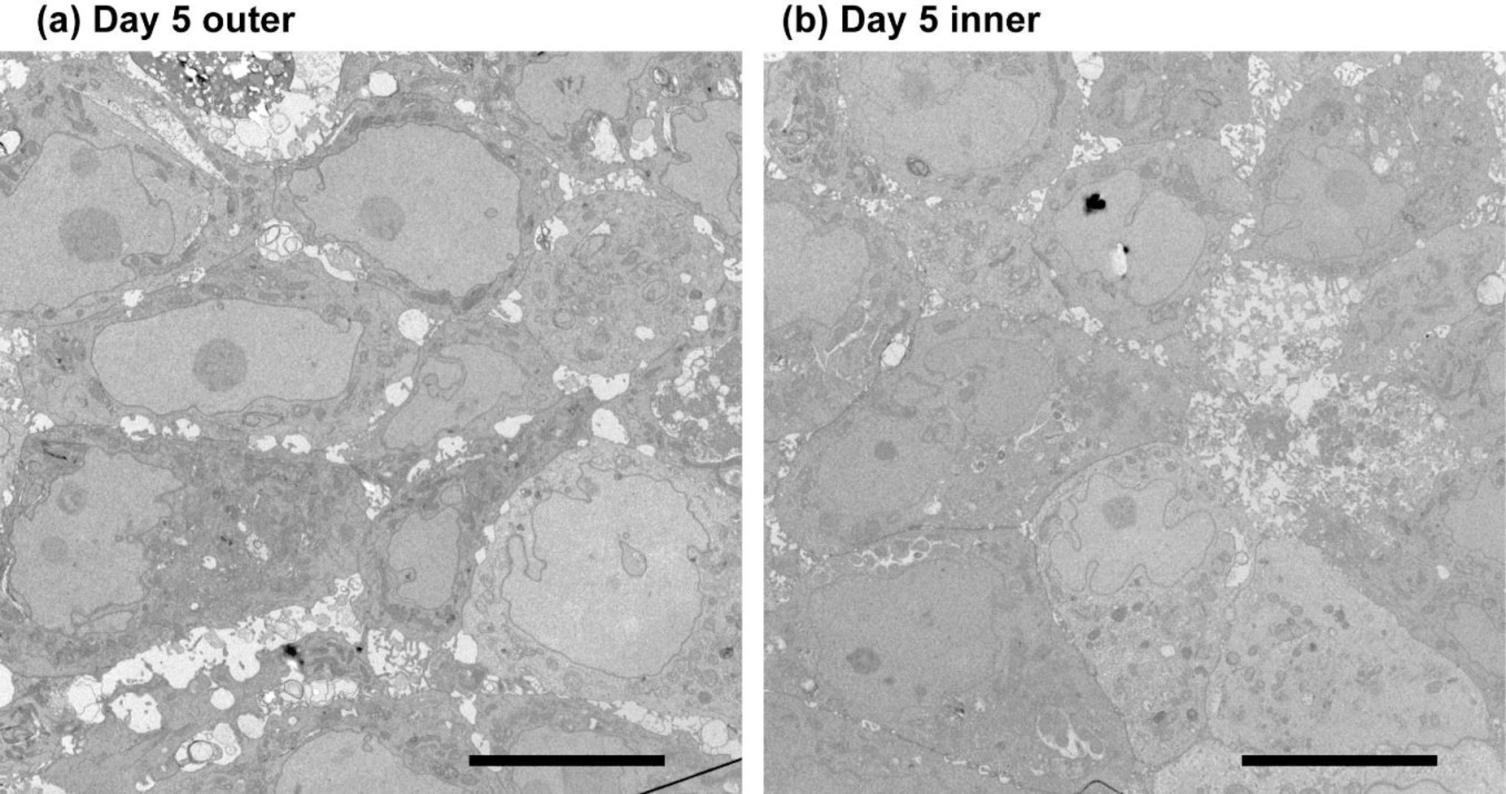
TEM images of a day 5 spheroid. Cells in (a) outer and (b) inner regions show similar structures. Bar = 10 micrometers.

**Fig 4:**
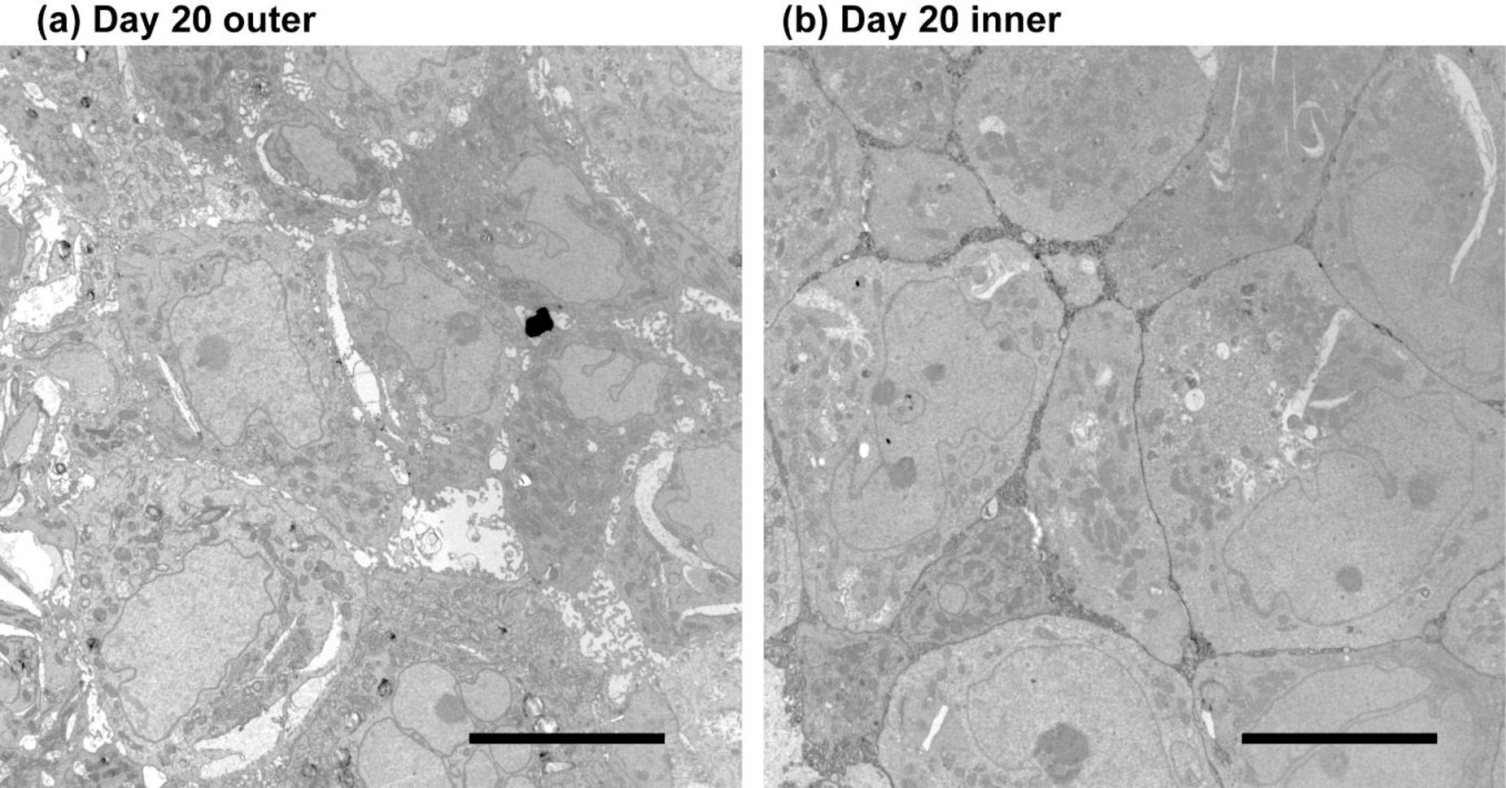
TEM images of a day 20 spheroid. Cells in (a) outer and (b) inner regions are compared. Inner regions show larger, closely-packed cells. Bar = 10 micrometers.

TEM analysis allows for the observation of the nanoscale cellular structures involved in cell-cell physical interactions at a resolution beyond that of optical microscopy [20]. Figs 3 and 4 are TEM images of typical inner and outer regions taken from a Day 20 spheroid and a Day 5 spheroid, respectively. Cells in the outer regions (i.e., areas close to the surfaces) show similar structures for Day 20 and 5 spheroids. Typically, there is an existence of intercellular spaces in these areas, observed as circular spots aligned along the boundary neighboring cells. On the other hand, cells in the inner regions of Day 20 spheroids tend to be larger and closely packed with tighter spacing (see Fig 4(B)). The difference between inner and outer regions is not obvious for day 5 spheroids. Although partial formation of tight junctions may be observed, intercellular spaces are still found in all regions of Day 5 spheroids.

**Fig 5:**
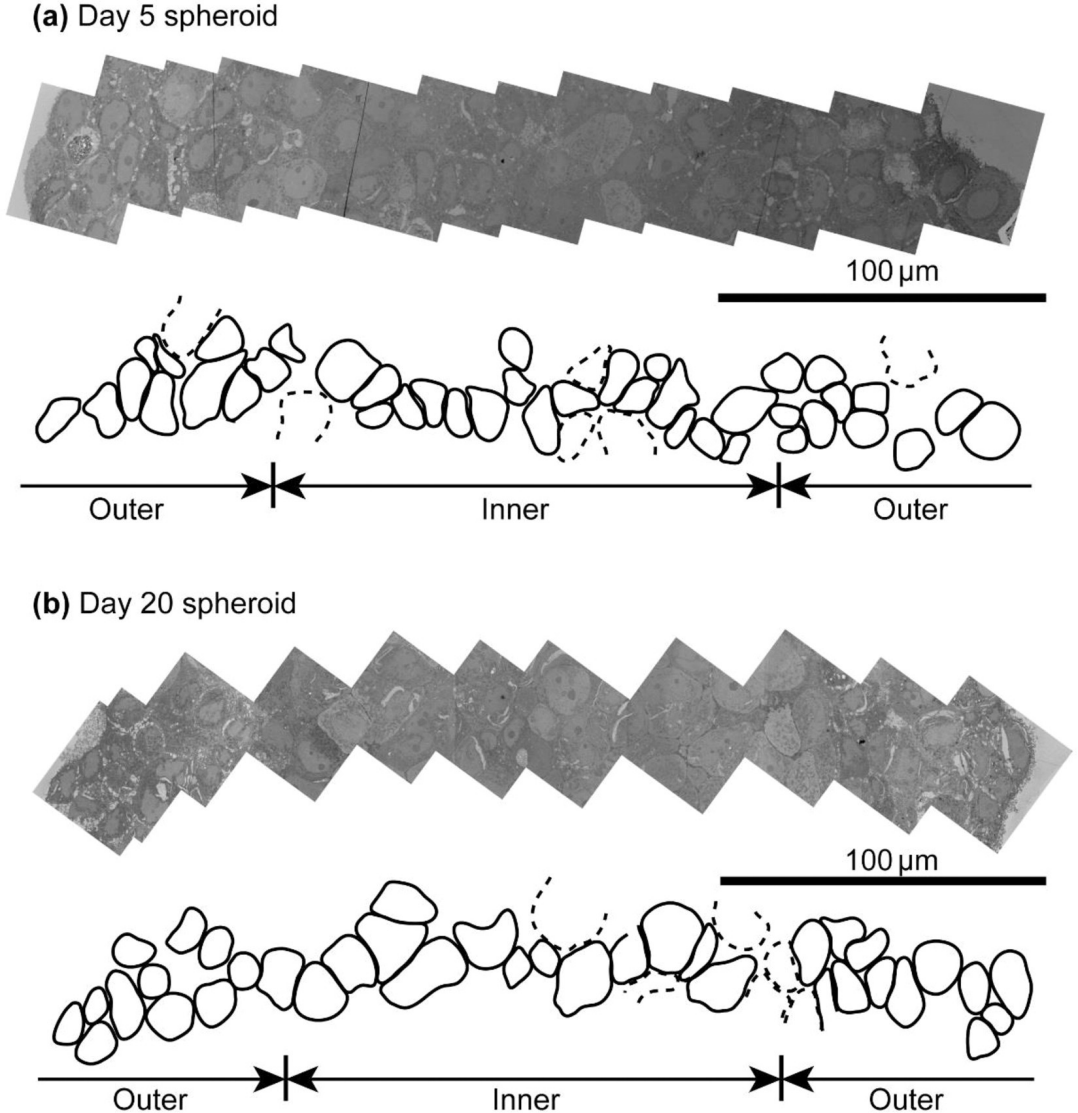
TEM images stitched along the long axes of spheroids. Manually traced shapes of cells. Cells indicated by dotted lines are incomplete cells only partially imaged.

The cell areas and spatial distribution were calculated from stitched high-resolution TEM images. Fig 5 shows examples of stitched images from a Day 5 and a Day 20 spheroid. Three spheroids were prepared and imaged for each of Day 5 and Day 20 conditions. The average cell and nucleus areas were calculated by dividing cells into two groups, namely, the outer 25% on both sides and the inner 50% as illustrated in Fig 5. Since the TEM specimens were prepared as a 2D slice of a 3D structure, some of the cells that were only partially included in the slice did not correctly represent their true dimensions. We counted only cells that showed the complete nucleus and the outside rim. Cells that did not show the entire section (i.e., cells partially cut out of the image) were omitted.

Areas of intercellular spaces were quantified through a custom MATLAB program in the following protocol:

1. Panels of TEM images were chosen for each spheroid,
  - The panel that is closest to the center point was used as the ‘center’ image.
  - Two outermost panels on both sides that do not include any part of the spheroid edge (often second-to-outermost panels) were used as the ‘outer’ images.
2. Inter-cellular spaces were counted in the two steps
  - The outlines of the cells in each image were manually traced.
  - Inter-cellular spaces between the outlines are filled.
3. The area of total inter-cellular spaces divided by the size of the image was found as the density.

**Fig 6:**
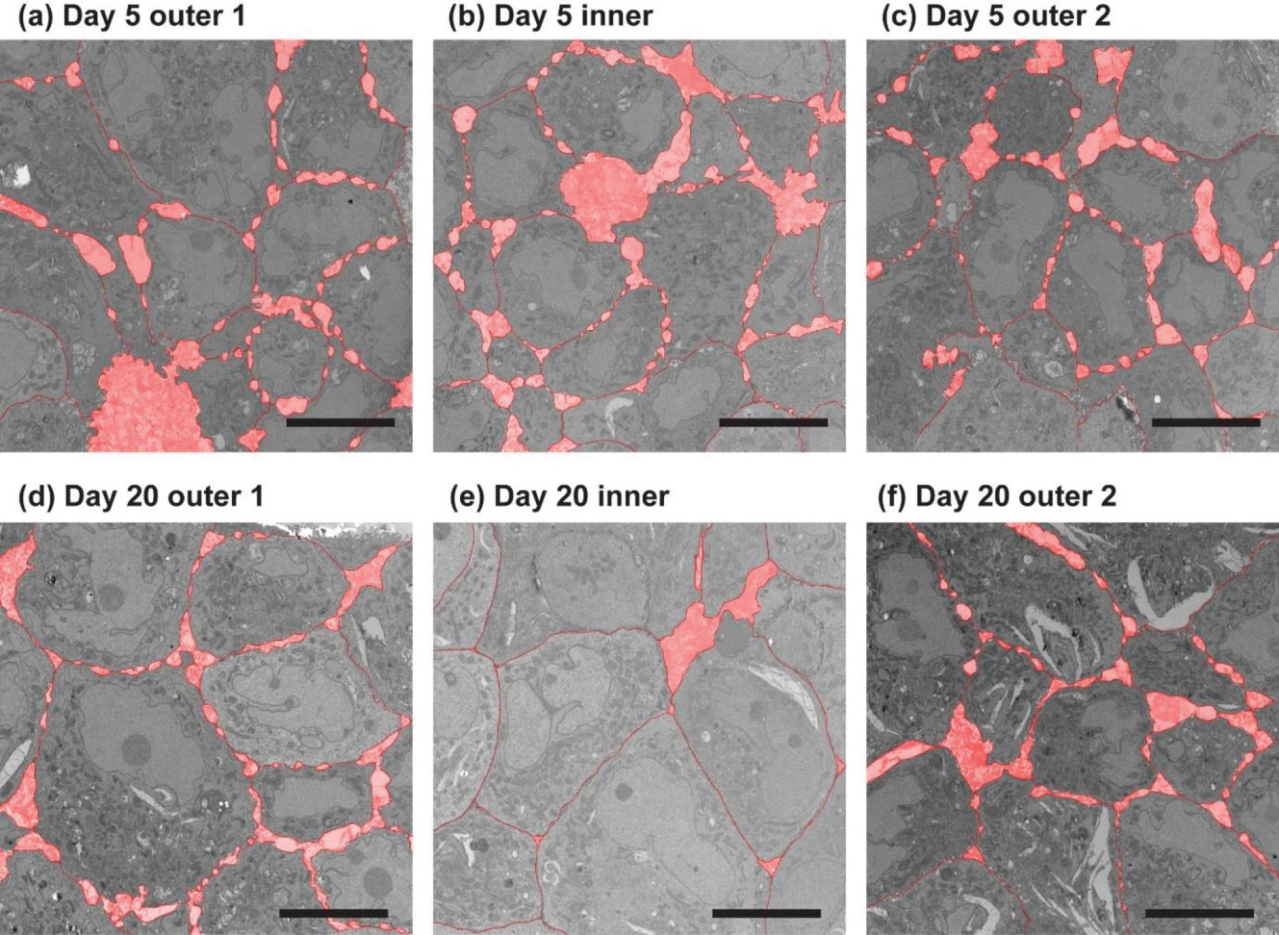
Analysis of a day 5 and day 20 spheroids TEM images. Red areas are the inter space regions selected through the image processing program. The more closed-packed and tighter inner structure of Day 20 spheroids has been quantified.

Examples of the processed images are shown in Fig 6(A)–6(F) for a Day 20 and a Day 5 spheroid, respectively. The traced lines are indicated by the dark red lines, and the inter-cellular spaces are filled with light red. As clearly visible in Fig 6(E), the inner regions of the Day 20 spheroids were closely packed with very small inter-cellular spaces, while the Day 5 spheroids show intercellular spaces with the density consistent throughout the spheroid.

The data analysis is summarized in Table 1. Cell and nucleus areas and the density of intercellular spaces in the inner regions of Day 20 spheroids were found to be significantly different from the outer regions, while no other areas showed a significant difference.

**Table 1:** 

		Cell (μm^2^)	Nucleus (μm^2^)	Inter-cellular space density(%)
Day 5 (3 spheroids)	Inner	123±53 (N = 62)	43±19 (N = 62)	12.5±3.0
	Outer	126±50 (N = 50)	41±18 (N = 50)	14.0±2.4
Day 20 (3 spheroids)	Inner	224±106 (N = 26)*	71±43 (N = 26)*	2.4±0.9**
	Outer	164±86 (N = 53)*	46±28 (N = 53)*	10.5±1.6**

Inner cores and outer regions are significantly different (p<0.005)*, (p<0.001)**.

**Fig 7:**
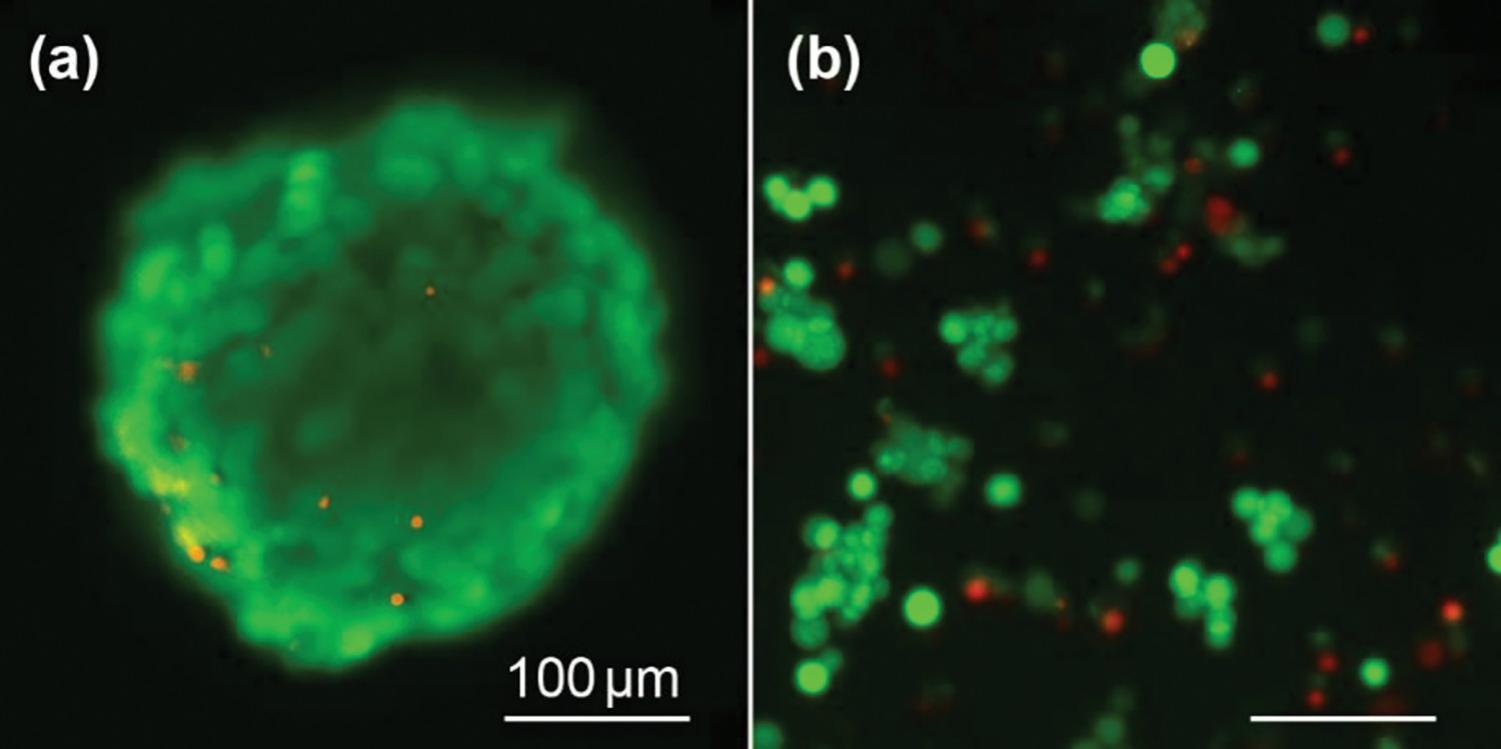
Viability assay of day 20 spheroids. Dispersed cells showed 18% dead cells.

The TEM images indicated that cell structures are intact within the central region, and the nuclei maintain an apparent complete nuclear chromatin density at 20 days. Given the possibility that the dense central core may be dead/necrotic cells, we imaged whole spheres with live-dead staining and fluorescence microscopy. Fig 7 shows an example of cell viability staining of spheroids at day 20 which is intact and mostly viable outside with less than 5% dead cells (Fig 7(A)). We also disrupted the sphere by trypsin digestion and labeled dispersed cells in a similar manner to observe the viability of cells within the inner region. Dispersed cells showed 18% dead cells, suggesting the inner region contains more dead cells (Fig 7(B)).

## 4. Discussion and conclusion

Due to the size gap between samples studied with atomic force microscopy and a tissue characterization apparatus, it has been difficult to quantify the mechanical characteristics of tumor spheroids. Conventionally, the formation of distinctive regions within a spheroid has been observed through light microscopy. In particular, the development of necrotic cores has been observed as an optically dark region in the center.

We have quantitatively compared the mechanical elasticities and internal structures of day 5 and day 20 spheroids seeded from T47D breast tumor cell lines. The mechanical characterization using custom-built force sensitive cantilevers measured the elastic moduli of day 5 and day 20 spheroids to be 260±100 and 680±150 Pa, respectively. As the TEM images indicate, the cells in the central core were not lysed yet at day 20. The elastic moduli were evaluated based on the assumption that a spheroid is a homogeneous spherical object. Vlashi et al. compared glucose uptake in breast tumor spheroids and reported that T47D showed a lower uptake than more aggressive MDAMB231 [30]. The lower oxygen demand of T47D spheroids is believed to have delayed the formation of a necrotic core compared to MDAMB231 spheroids studied in [23].

Furthermore, we used transmission electron microscopy (TEM) to image ultrathin (~ 100 nm) sections of tumor spheroids. These images were analyzed to measure the cell and nuclear areas in different regions. As spheroids grow older, they develop an optically dark region in the center (see Fig 1). This dark region made it difficult to use fluorescence imaging for a quantitative comparative study. Fluorescence intensities observed in day 20 spheroids were much weaker than those from day 5 spheroids. TEM imaging of cryosectioned samples allowed for quantitative morphological characterization regardless of the growth stages, thus avoiding imaging artifacts.

Through the TEM analysis, we found that the cells in the inner cores of day 20 spheroids are occupying areas significantly larger than cells in other areas. Interestingly, Delarue et al. reported the reduction of the cell volume and proliferation inhibition, both observed in the core of the spheroid [31]. However, the former occurs on timescales of minutes, and the latter occurs on timescales of hours. The effect of pressure on the proliferation was antagonized by silencing the proliferation inhibitor, p27_Kip1_. On the other hand, silencing the inhibitor did affect the cell volume reduction, which suggested that the volume reduction was a passive, short-time sponge-like response to accommodate the volume [31], while the proliferation inhibition was a separate event induced by volume reduction. We observed the cell volume increase on an even longer timescale of 20 days. We speculate that in our experiment, the proliferation inhibition observed at the spheroid plateau stage, appeared to increase the volume of individual cells and the cell nuclei. Thus, it is clear that cell volume changes in spheroids over time are affected by multiple influences including intra-spheroid pressure, proliferation, and metabolism.

Also, we observed that the cells in the inner regions of day 5 spheroids lacked closed packed cellular region and did not show significant differences from cells in outer regions. The internal cellular assembly of the spheroid was also studied, and it was found that the cells in the inner part of the day 20 spheroids were closely fit and showed very small intercellular spaces. In contrast, other cells showed bead-shaped interstitial spaces between adjacent cells.

In conclusion, older spheroids form an inner core composed of larger cells with reduced interstitial spaces. The observed reduction of the interstitial space may be one factor that contributes to stiffer older spheroids.

## Supporting information

S1 File This file shows the analysis of all TEM images we used for the image analysis. (PDF)
