# Supplementary material for: Electron microscopy imaging and mechanical characterization of T47D multicellular tumor spheroids–Older spheroids reduce interstitial space and become stiffer
Source: PLoS One. 2023 May 25;18(5):e0286291. doi: 10.1371/journal.pone.0286291 (PMC10212087; doi:10.1371/journal.pone.0286291)
Supplement: S1 File — (PDF) [file pone.0286291.s001.pdf]

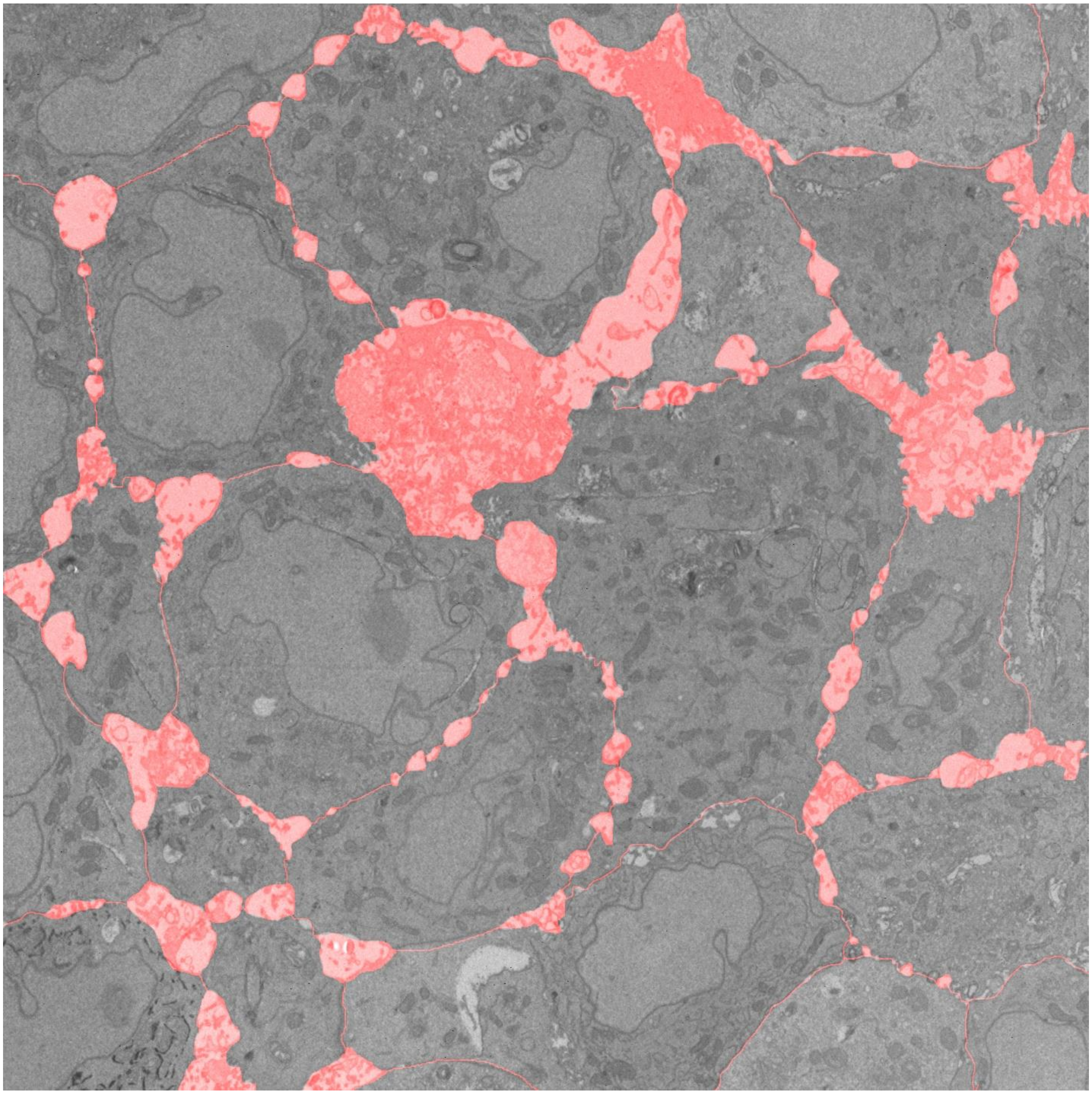

Day 5 sample 1 inner

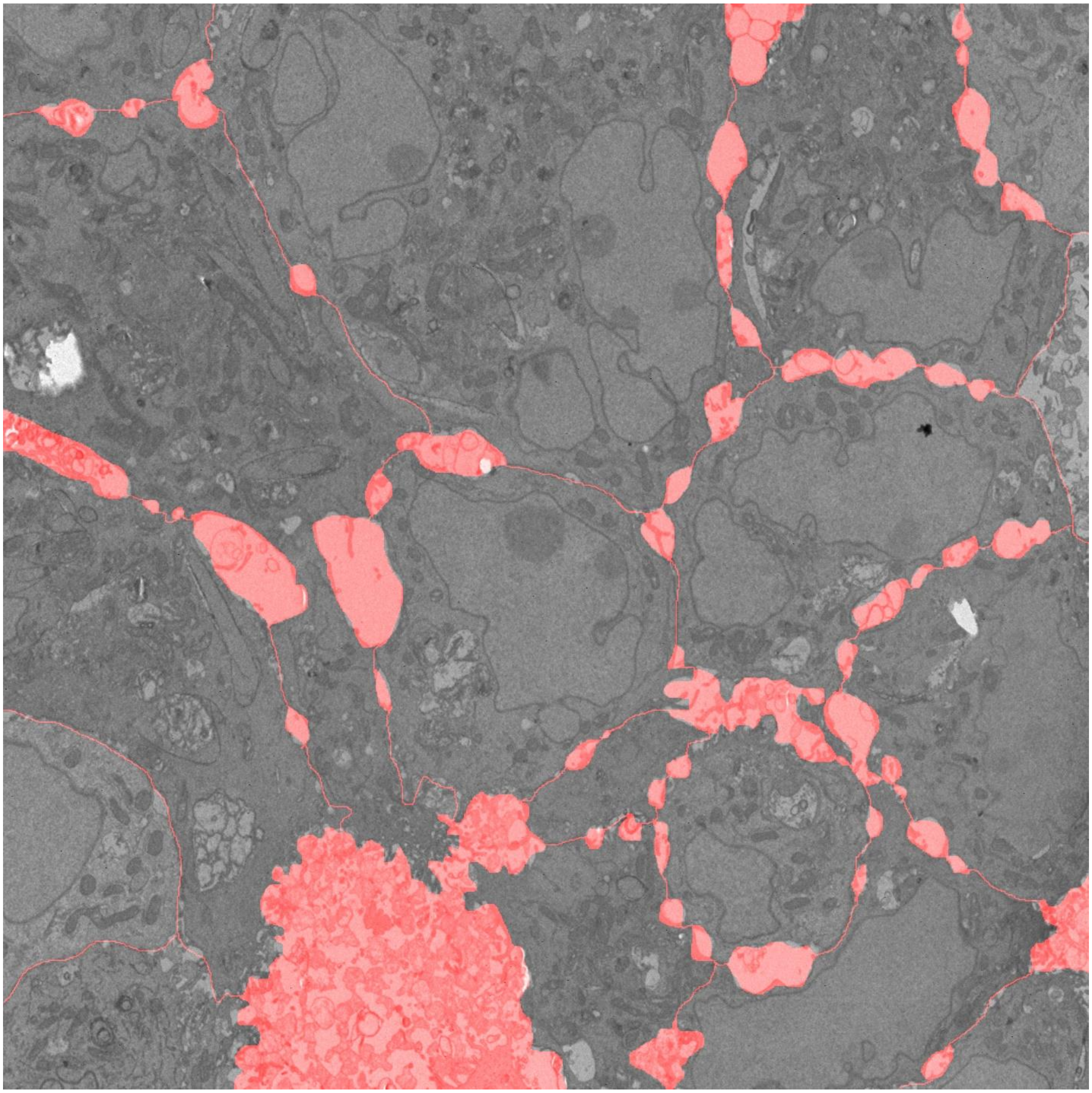

Day 5 sample 1 outer1

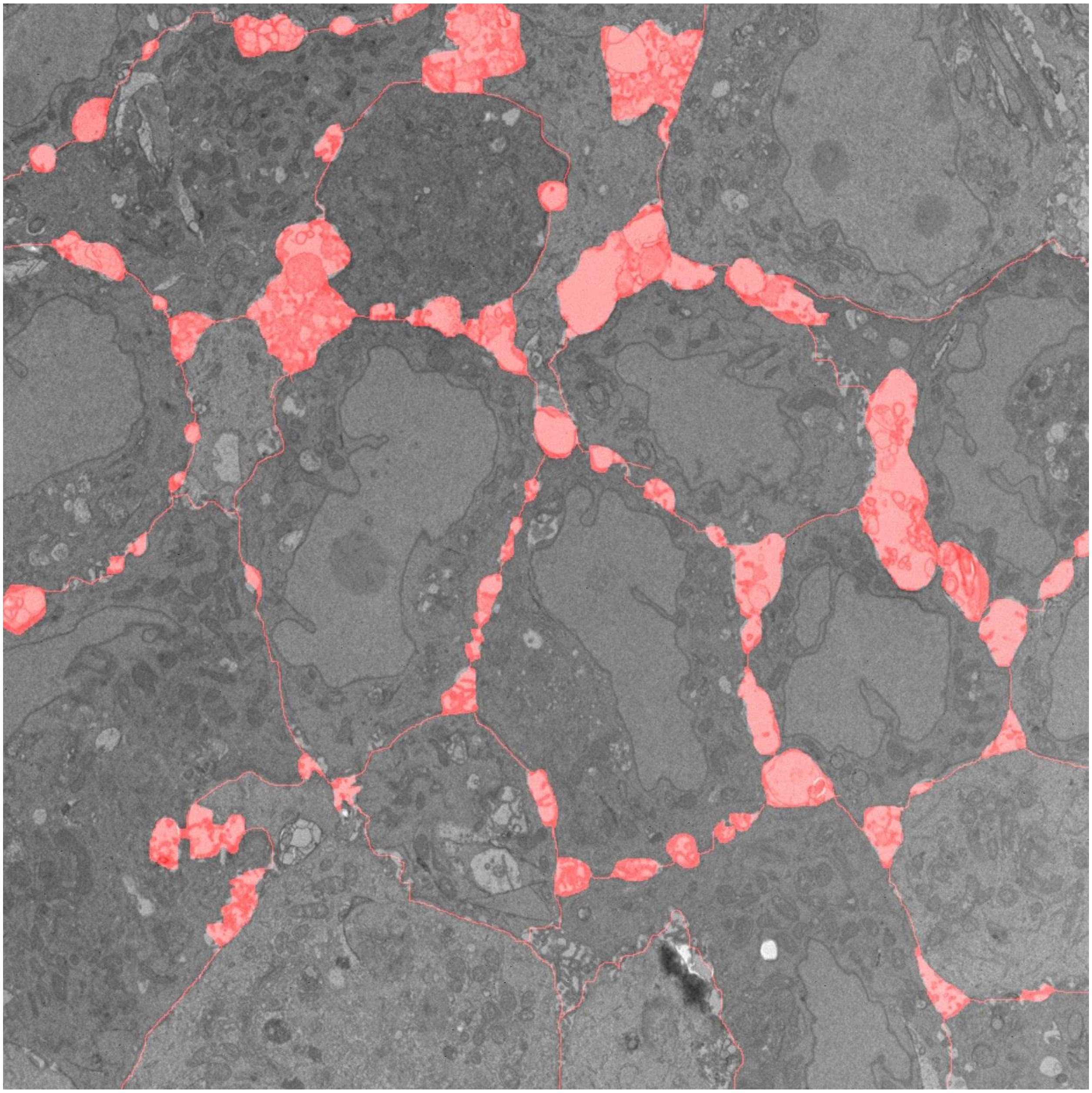

Day 5 sample 1 outer2

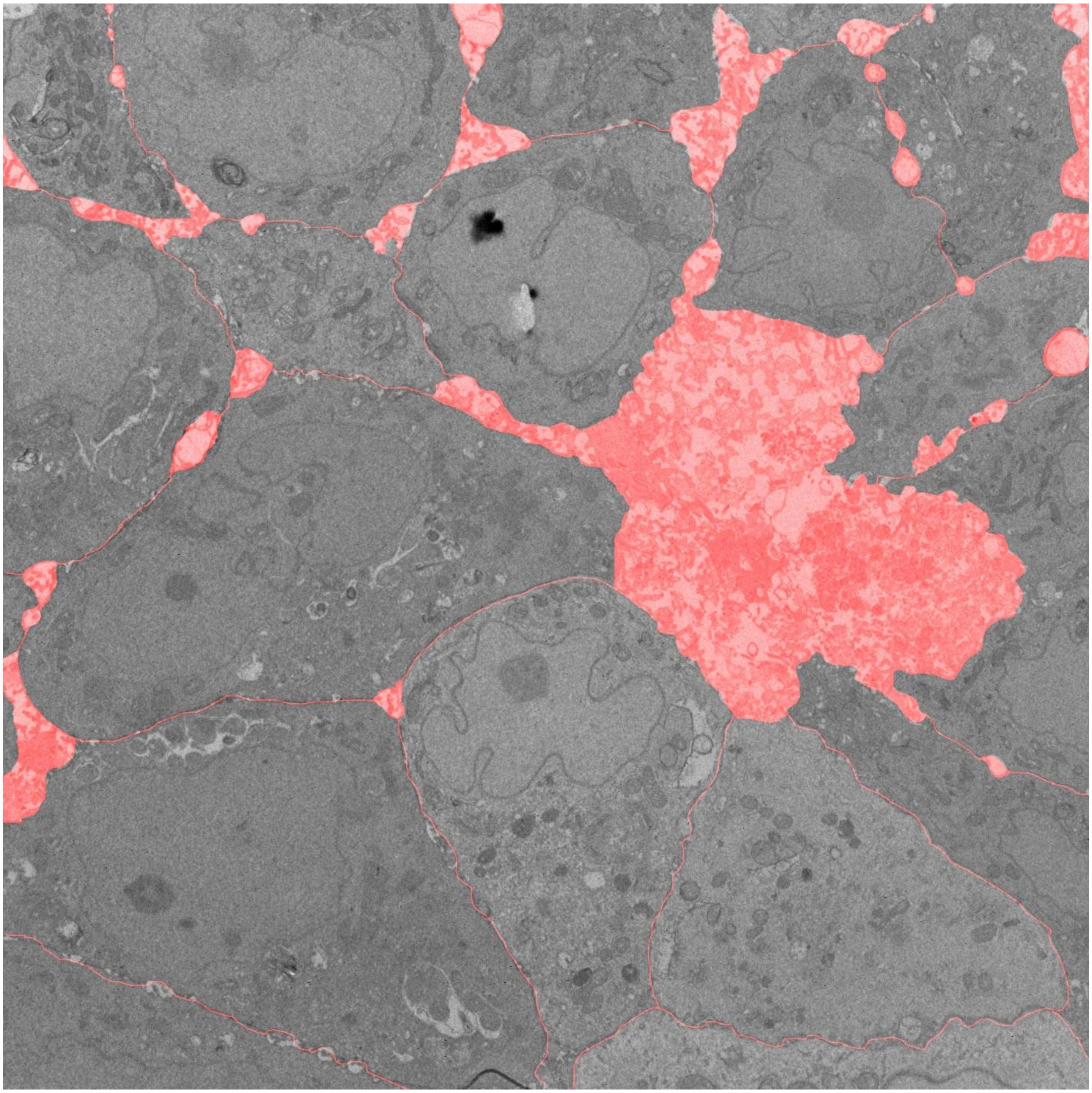

Day 5 sample 2 inner

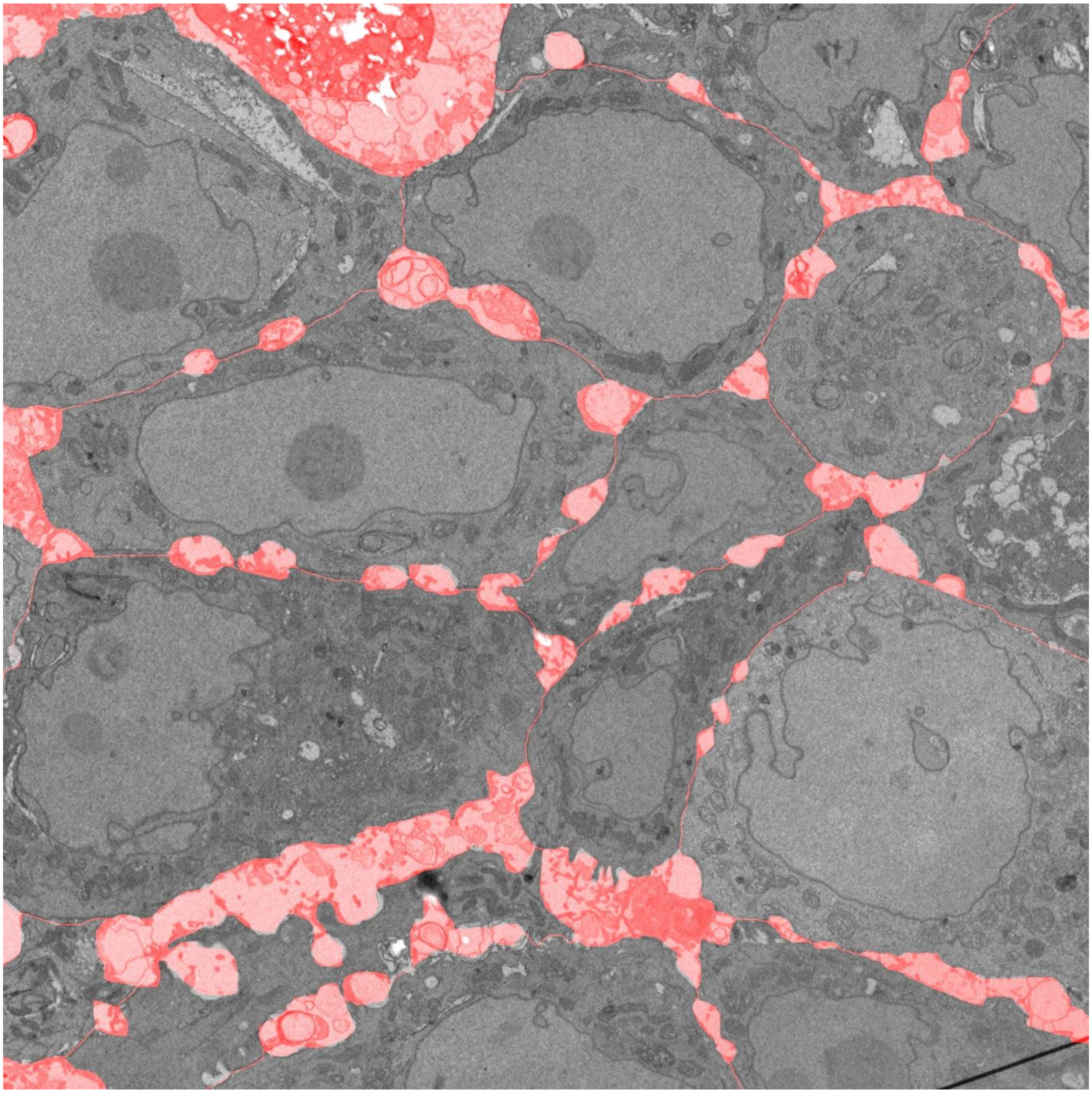

Day 5 sample 2 outer1

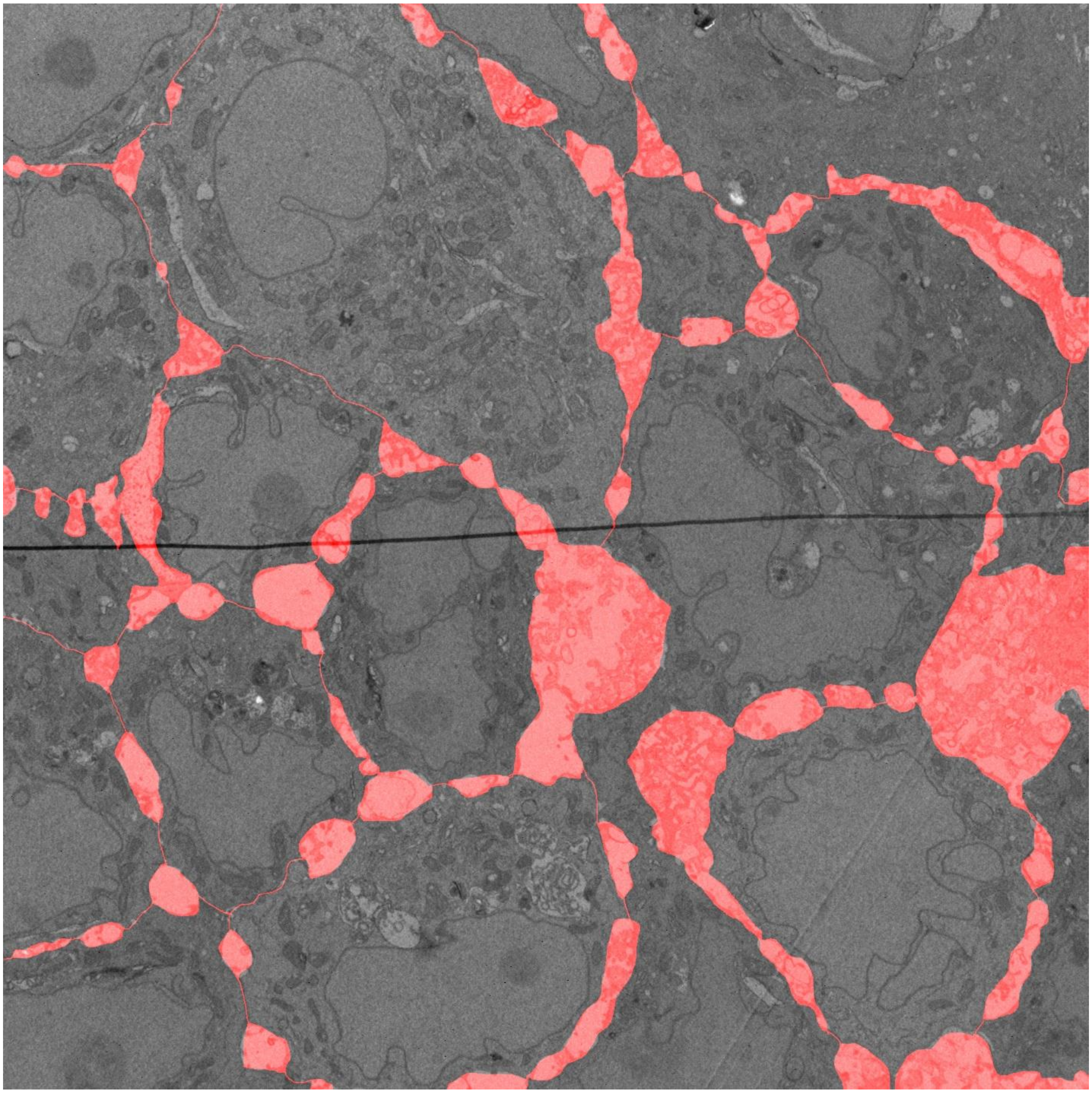

Day 5 sample 2 outer2

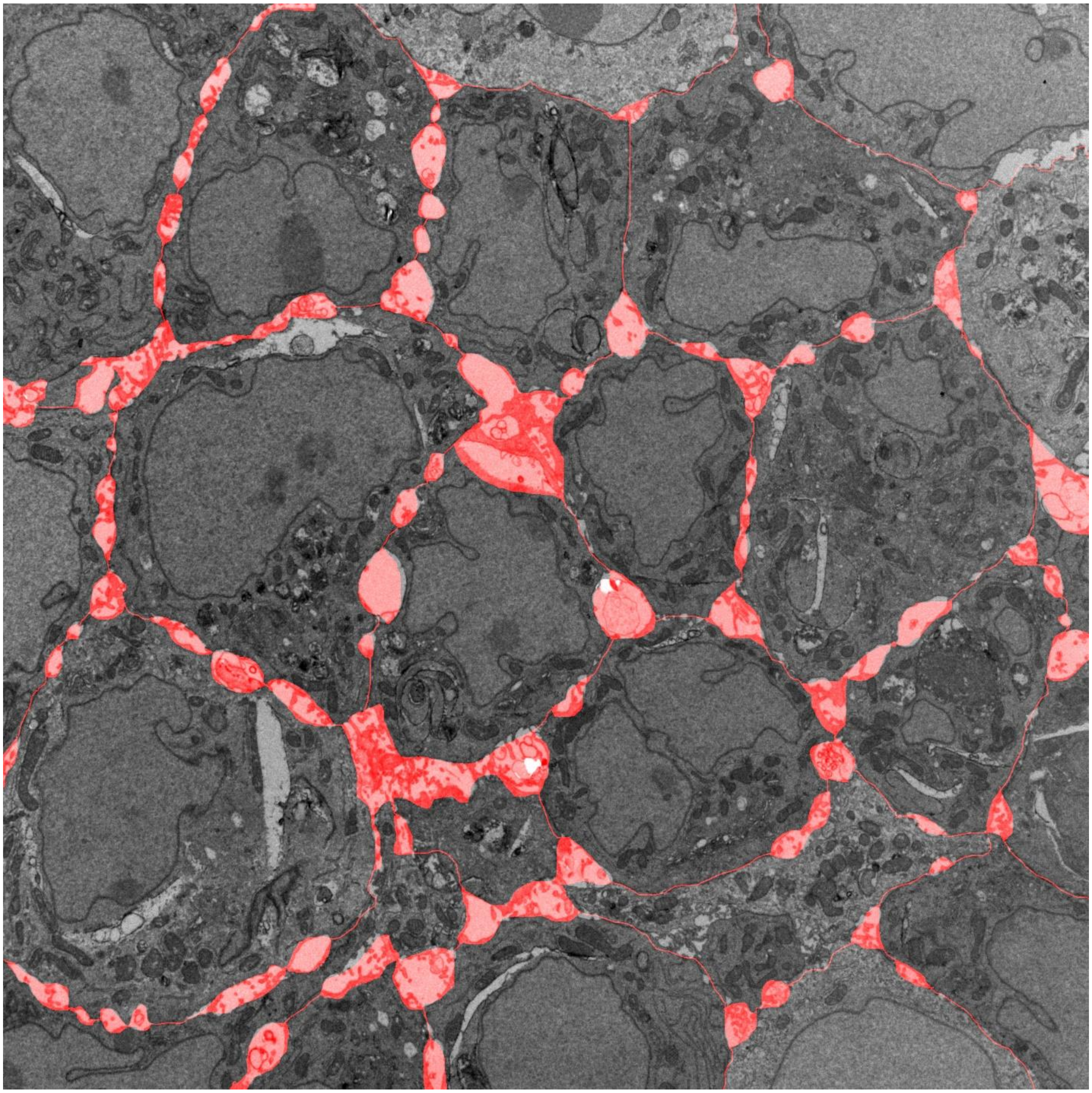

Day 5 sample 3 inner

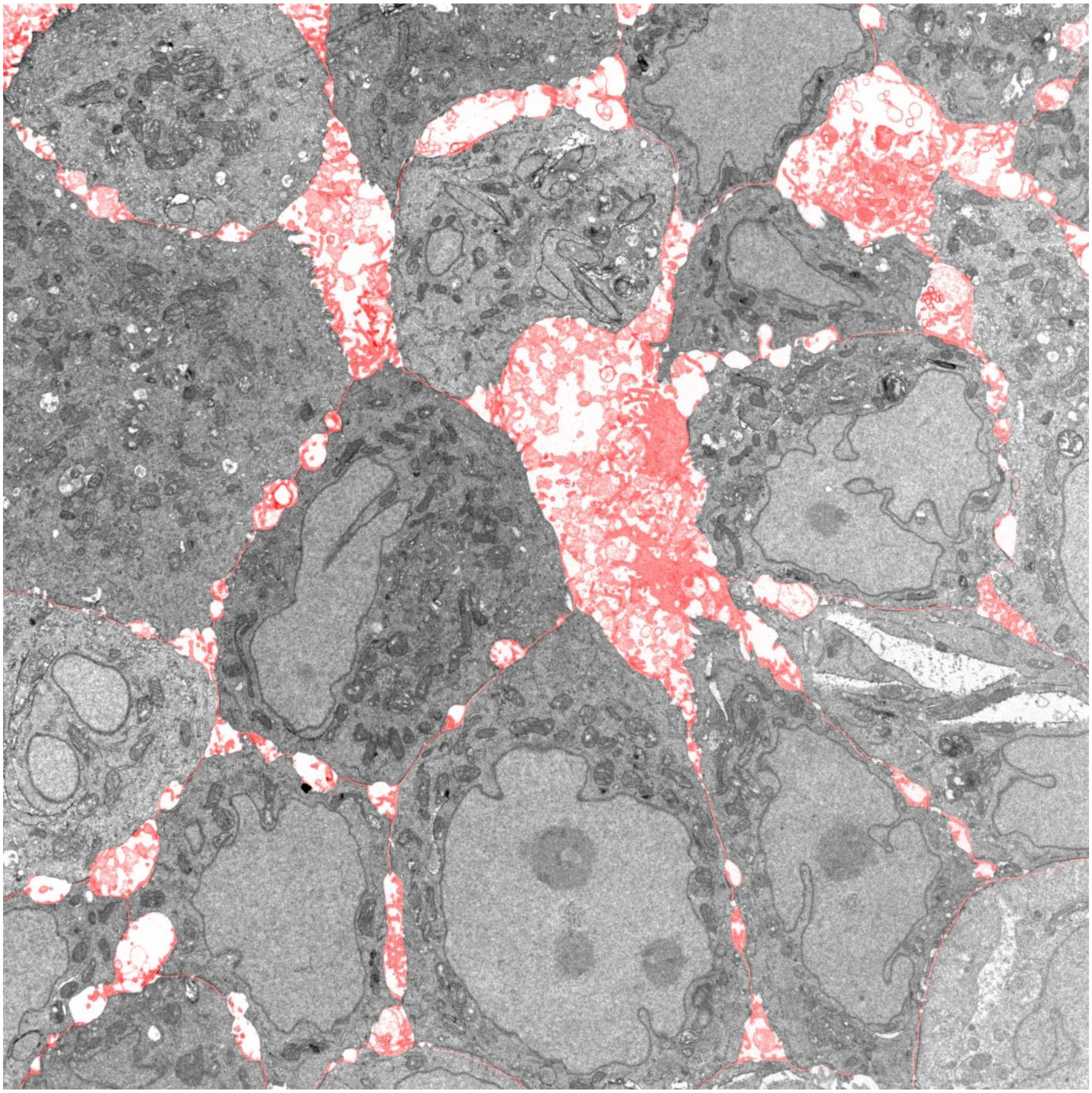

Day 5 sample 3 outer1

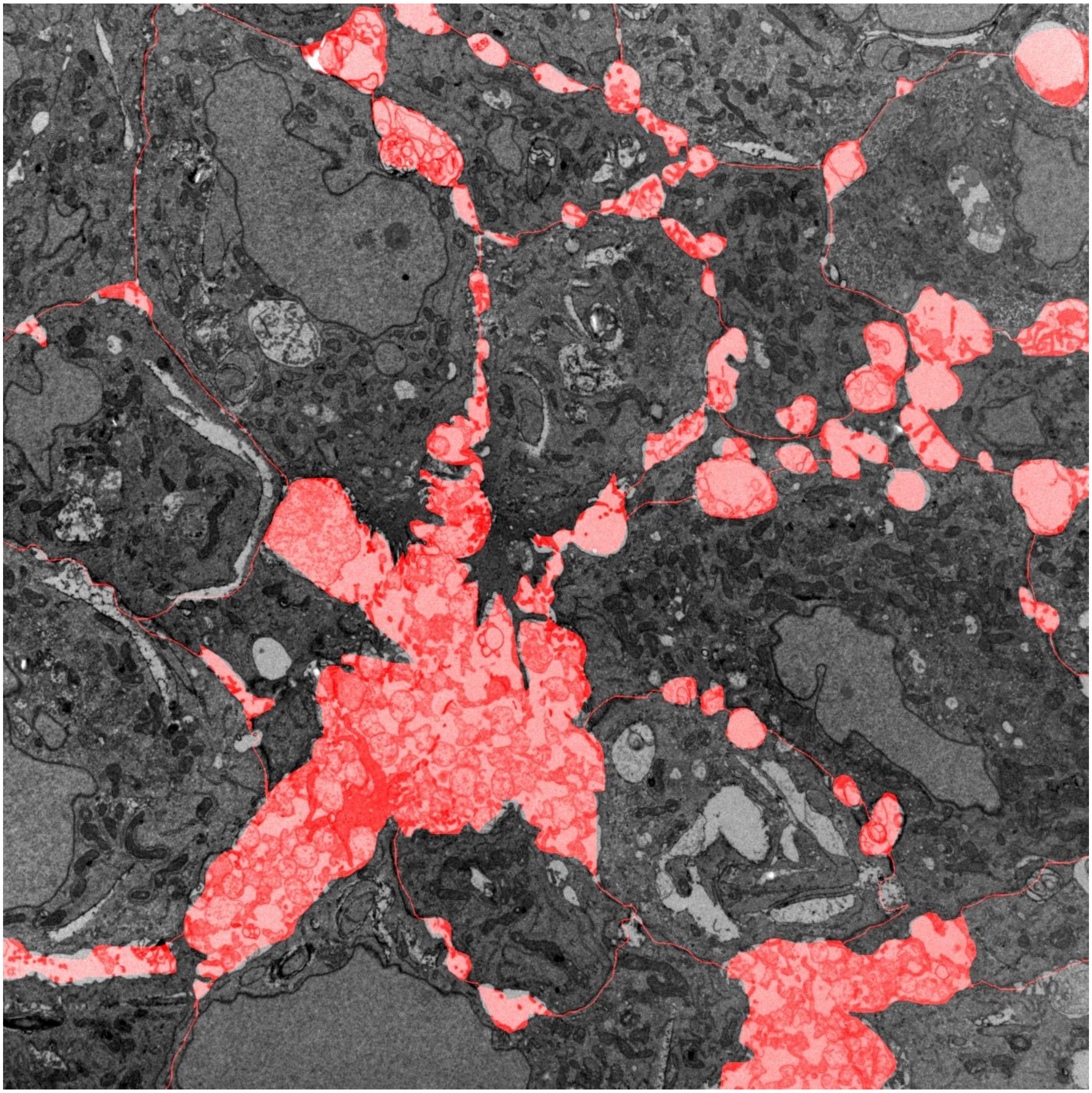

Day 5 sample 3 outer2

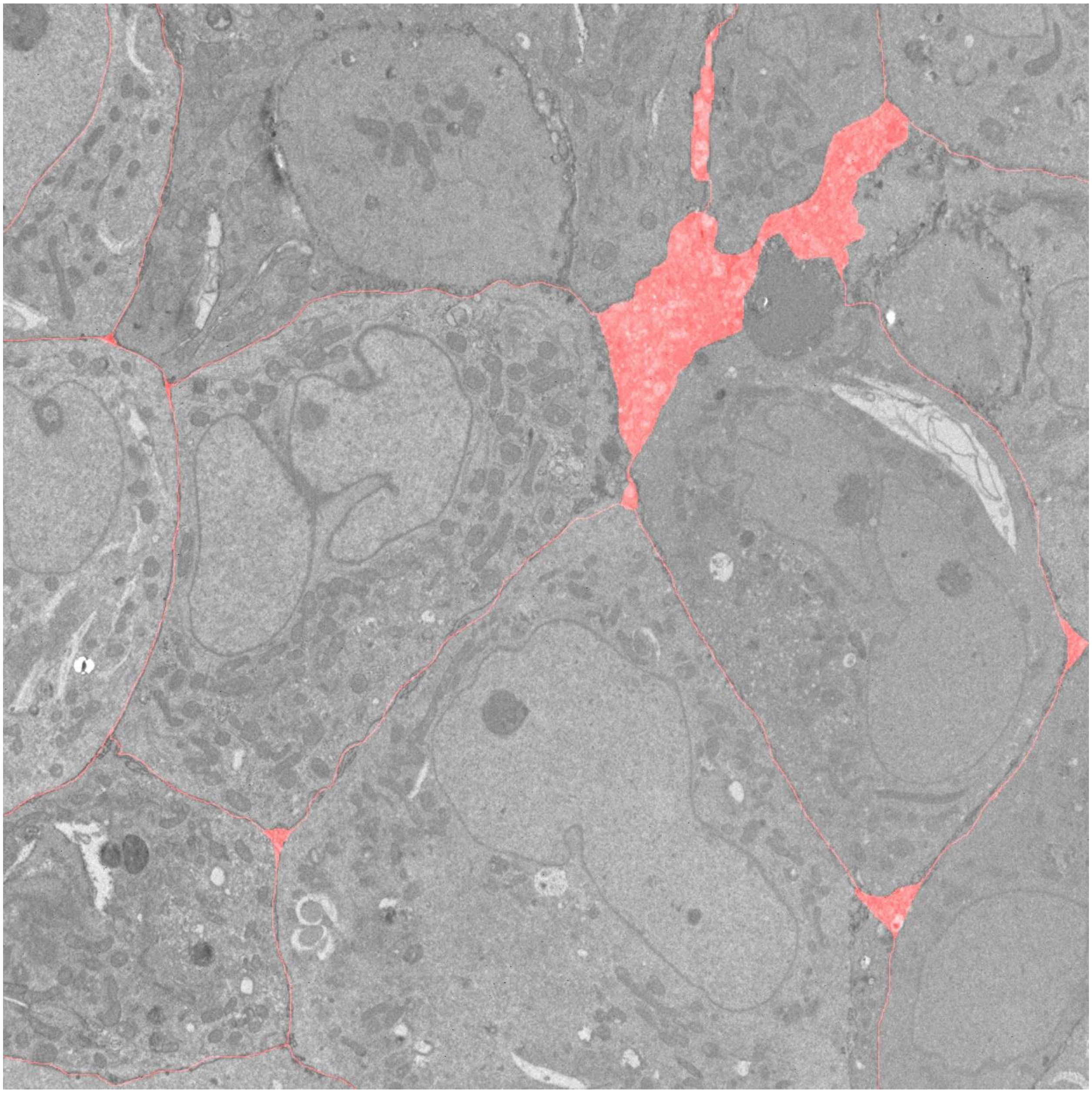

Day 20 sample 1 inner

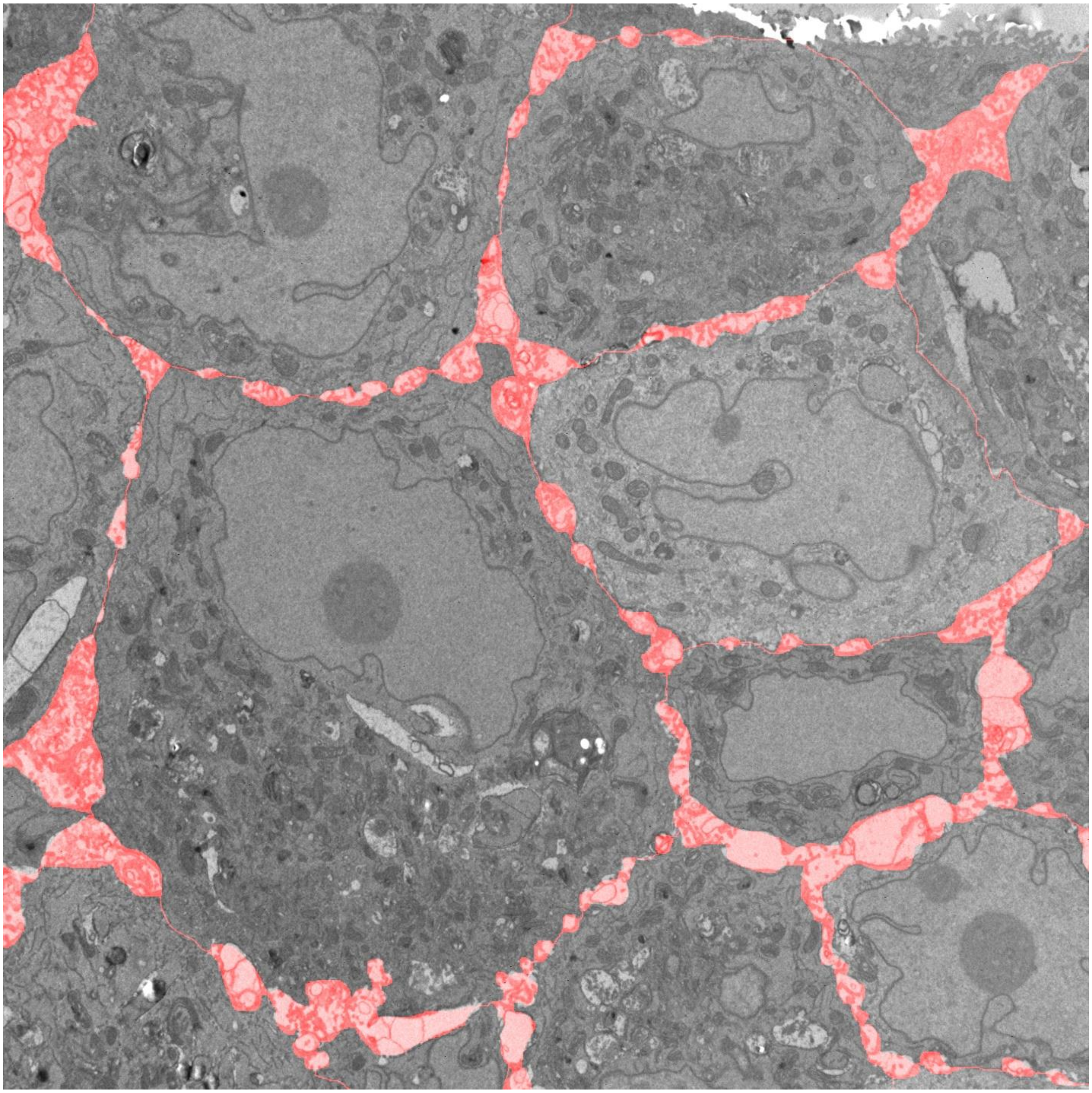

Day 20 sample 1 outer1

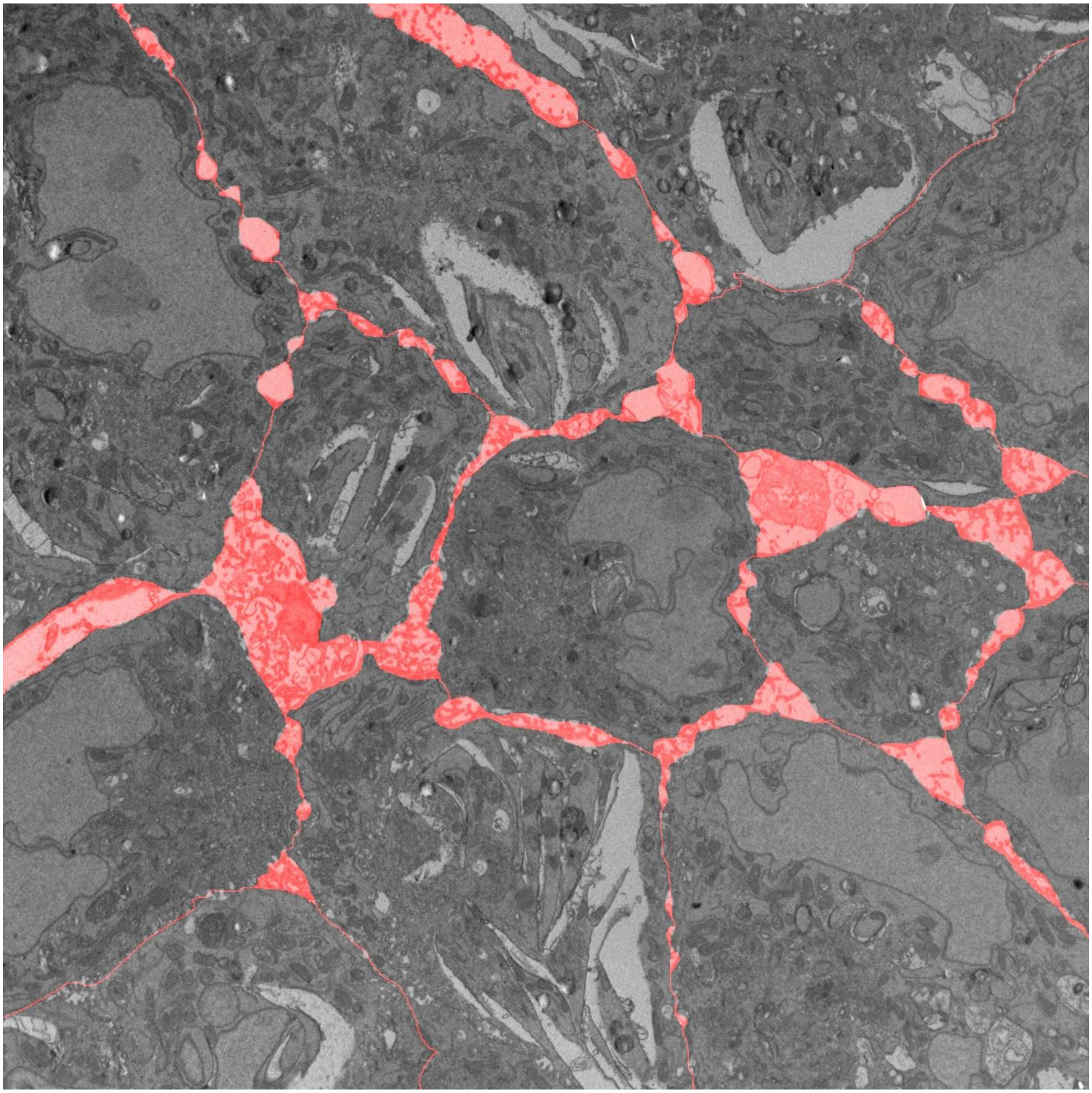

Day 20 sample 1 outer2

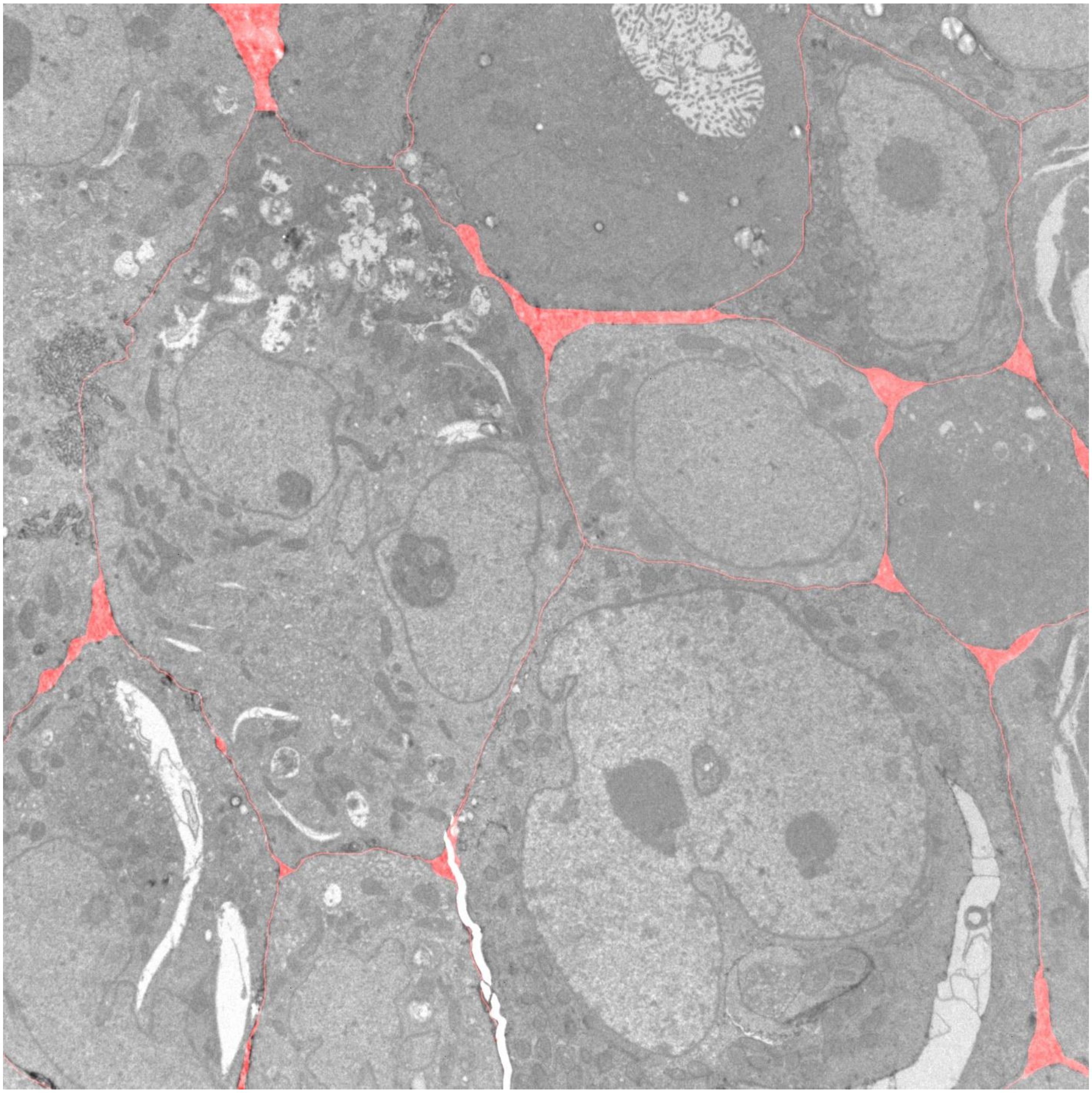

Day 20 sample 2 inner

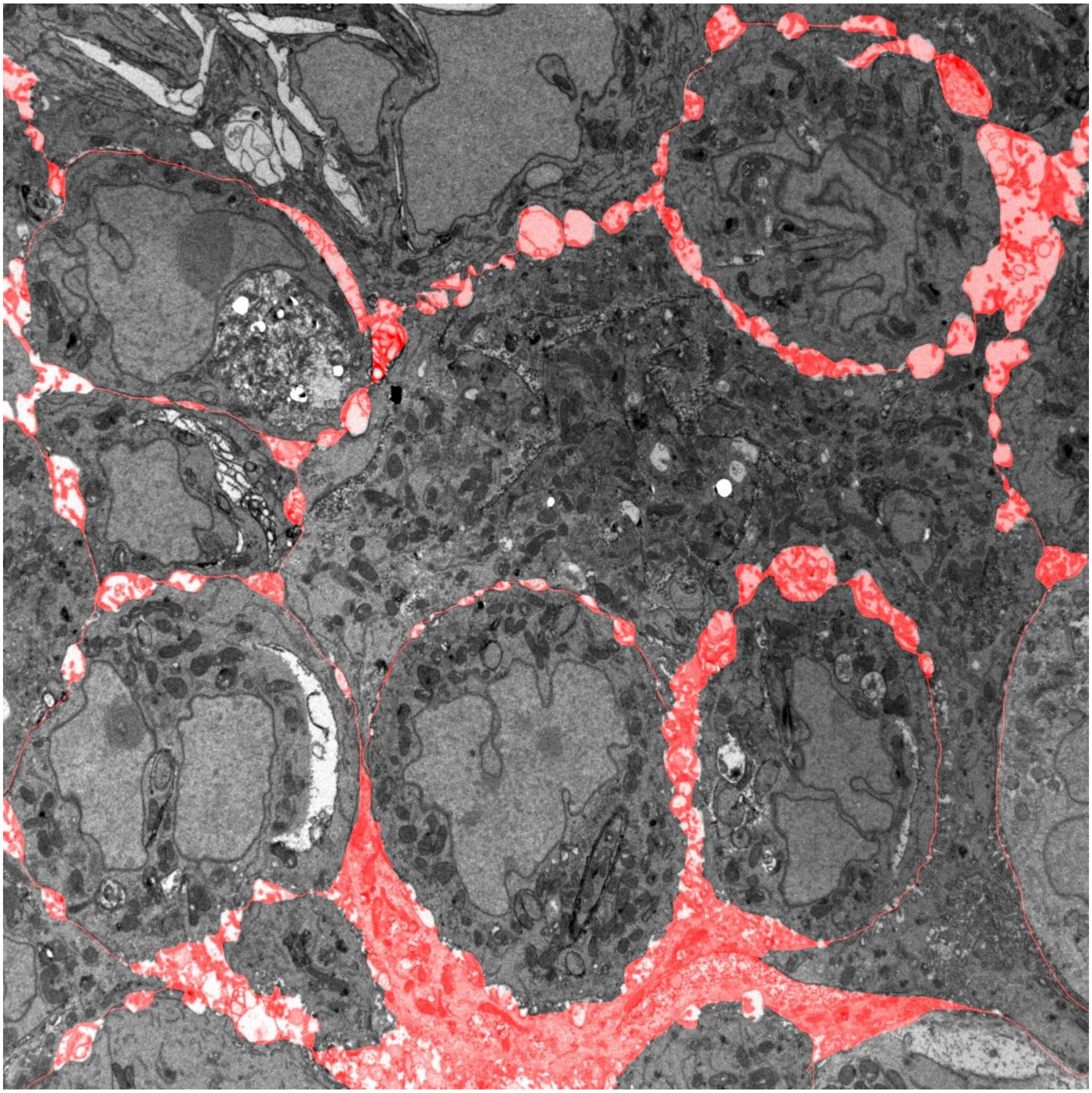

Day 20 sample 2 outer1

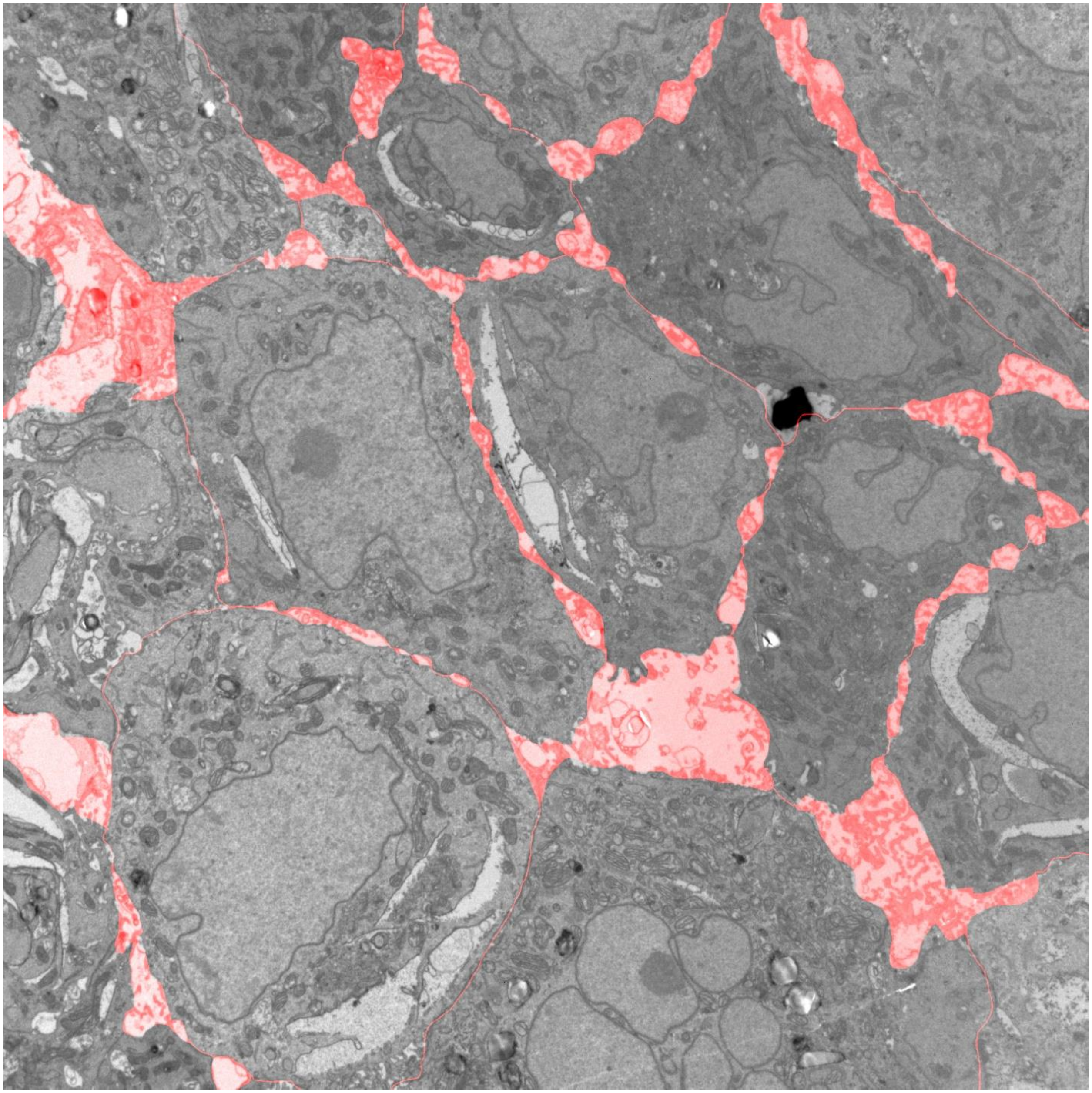

Day 20 sample 2 outer2

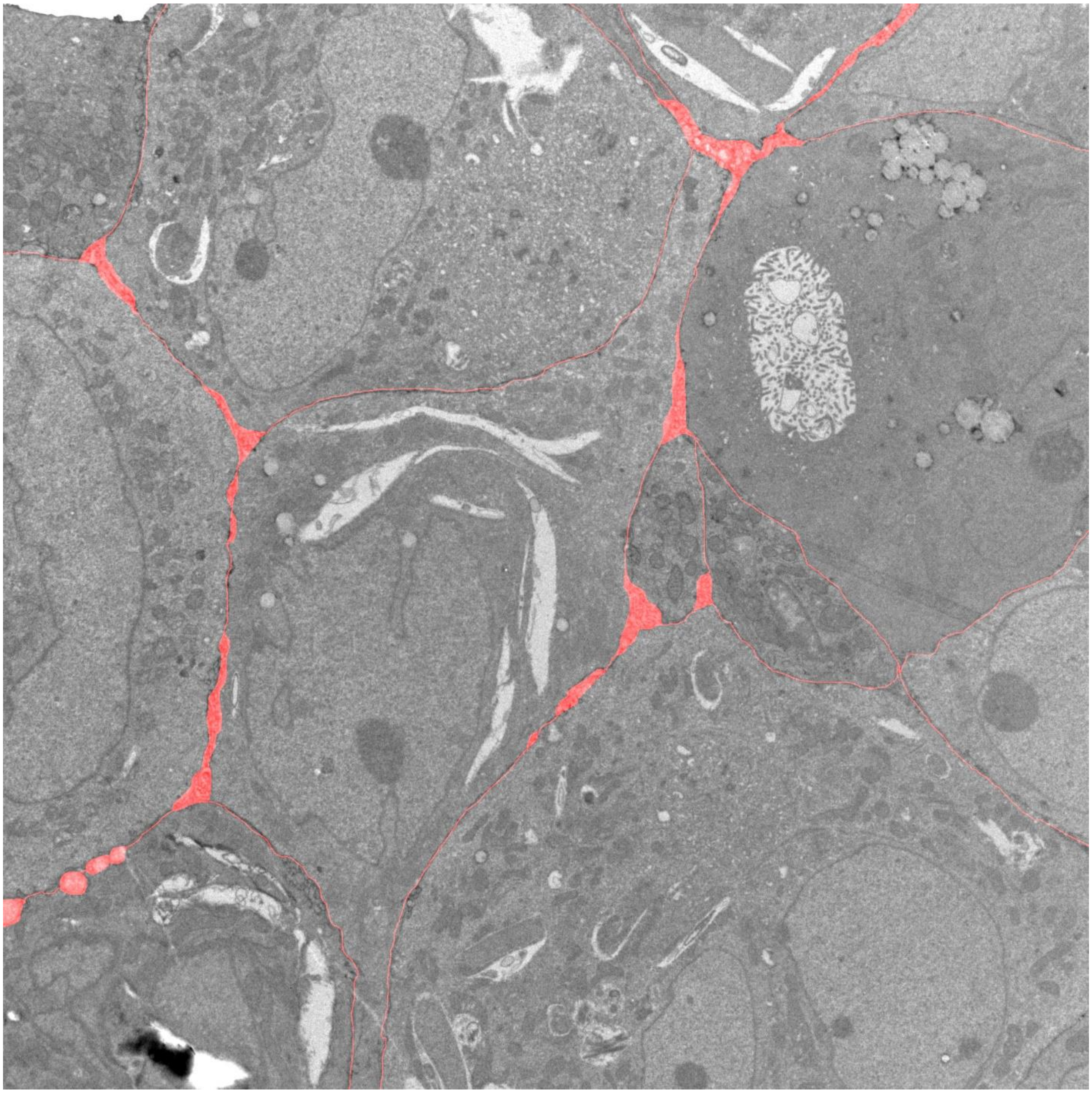

Day 20 sample 3 inner

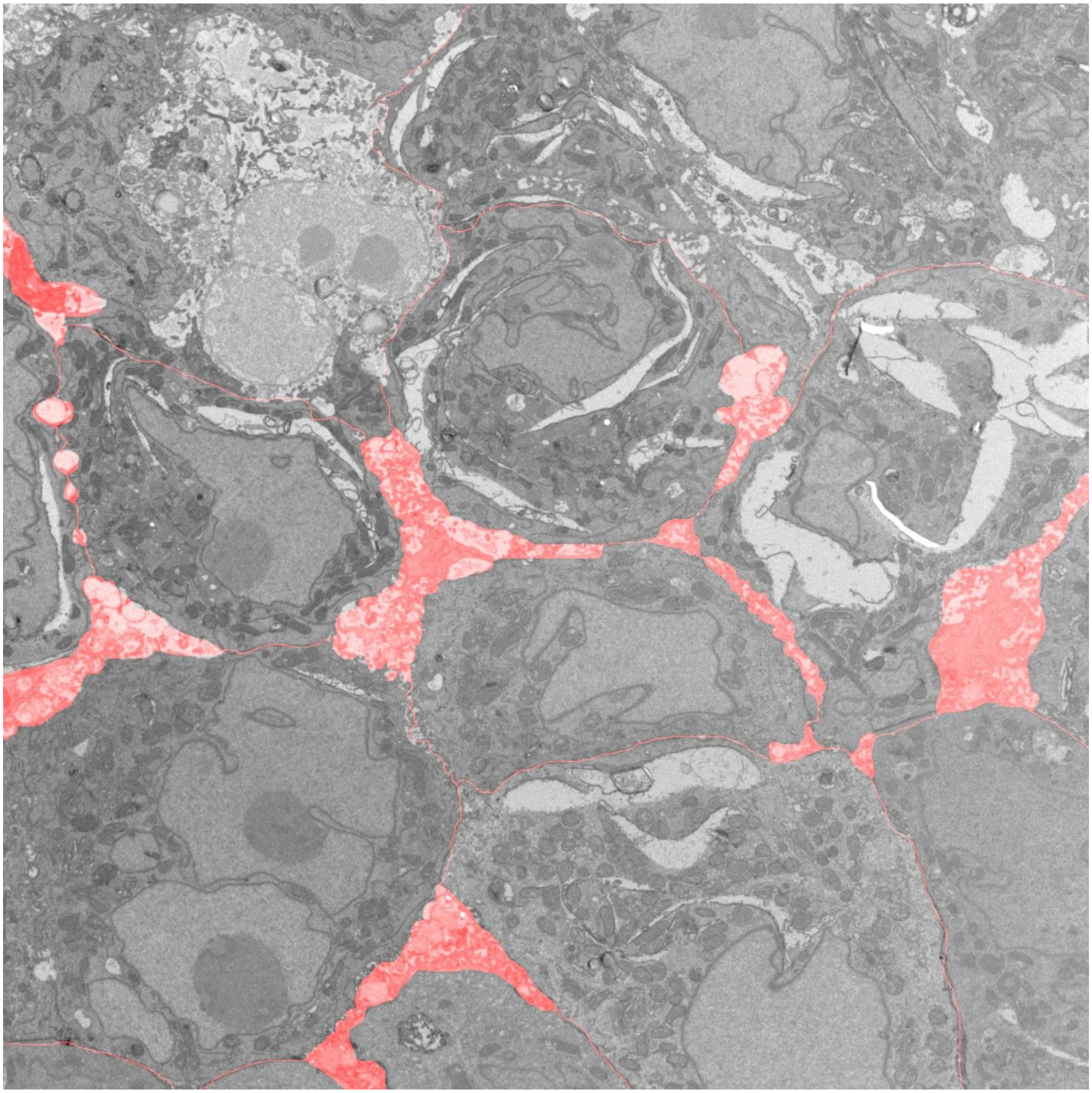

Day 20 sample 3 outer1

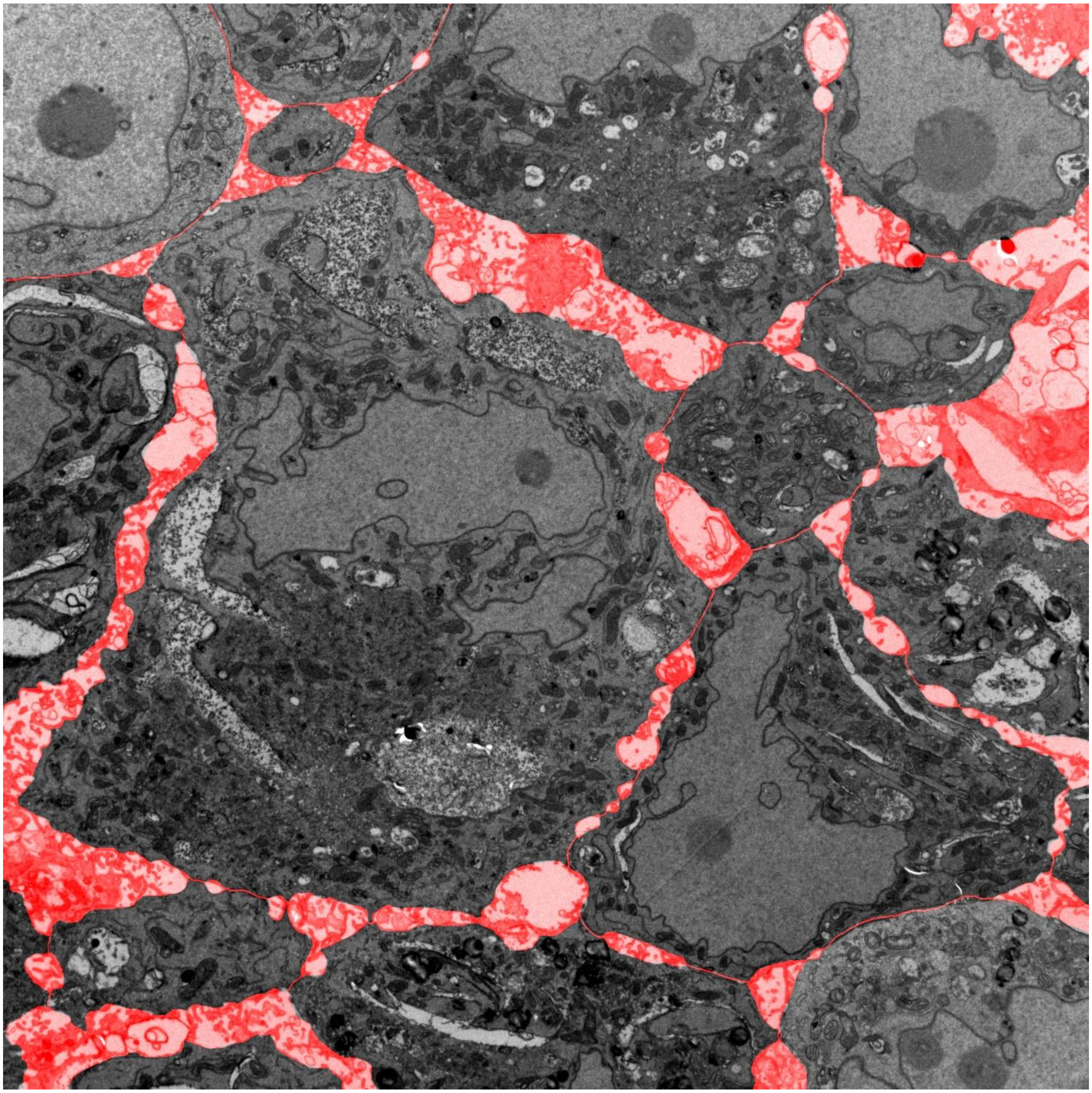

Day 20 sample 3 outer2
